# Pseudomonas aeruginosa Alters Its Transcriptome Related to Carbon Metabolism and Virulence as a Possible Survival Strategy in Blood from Trauma Patients

**DOI:** 10.1128/mSystems.00312-18

**Published:** 2019-05-07

**Authors:** Moamen M. Elmassry, Nithya S. Mudaliar, Kameswara Rao Kottapalli, Sharmila Dissanaike, John A. Griswold, Michael J. San Francisco, Jane A. Colmer-Hamood, Abdul N. Hamood

**Affiliations:** a Department of Biological Sciences, Texas Tech University, Lubbock, Texas, USA; b Department of Surgery, Texas Tech University Health Sciences Center, Lubbock, Texas, USA; c Center for Biotechnology and Genomics, Texas Tech University, Lubbock, Texas, USA; d Honors College, Texas Tech University, Lubbock, Texas, USA; e Department of Immunology and Molecular Microbiology, Texas Tech University Health Sciences Center, Lubbock, Texas, USA; f Department of Medical Education, Texas Tech University Health Sciences Center, Lubbock, Texas, USA; University of Pennsylvania

**Keywords:** *Pseudomonas aeruginosa*, blood, metabolism, metabolome, sepsis, transcriptome, trauma, virulence

## Abstract

Trauma patients (TPs) are highly susceptible to infections, which often lead to sepsis. Among the numerous causative agents, Pseudomonas aeruginosa is especially important, as P. aeruginosa sepsis is often fatal. Understanding the mechanism of its pathogenesis in bloodstream infections is imperative; however, this mechanism has not been previously described. To examine the effect of trauma-induced changes in blood on the expression of P. aeruginosa genes, we grew strain UCBPP-PA14 (PA14) in blood samples from eight TPs and seven healthy volunteers (HVs). Compared with its growth in blood from HVs, the growth of PA14 in blood from TPs significantly altered the expression of 285 genes. Genes whose expression was significantly increased were related to carbon metabolism, especially malonate utilization and mannitol uptake, and efflux of heavy metals. Genes whose expression was significantly reduced included genes of the type VI secretion system, genes related to uptake and metabolism of amino acids, and genes related to biosynthesis and transport of the siderophores pyoverdine and pyochelin. These results suggest that during systemic infection in trauma patients, and to adapt to the trauma-induced changes in blood, P. aeruginosa adjusts positively and negatively the expression of numerous genes related to carbon metabolism and virulence, respectively.

**IMPORTANCE** While a considerable body of knowledge regarding sepsis in trauma patients is available, the potential influence of trauma-induced changes in the blood of these patients on the pathogenesis of Pseudomonas aeruginosa is basically an unexplored area. Rather than using standard laboratory media, we grew P. aeruginosa in whole blood from either healthy volunteers or trauma patients. The specific changes in the P. aeruginosa transcriptome in response to growth in blood from trauma patients reflect the adaptation of this organism to the bloodstream environment. This knowledge is vital for understanding the strategies this pathogen uses to adapt and survive within the host during systemic infection. Such information will help researchers and clinicians to develop new approaches for treatment of sepsis caused by P. aeruginosa in trauma patients, especially in terms of recognizing the effects of specific therapies (e.g., iron, zinc, or mannitol) on the organism. Further, this information can most likely be extrapolated to all patients with P. aeruginosa septicemia.

**Author Video**: An author video summary of this article is available.

## Abstract (precis)

While a considerable body of knowledge regarding sepsis in trauma patients is available, the potential influence of trauma-induced changes in the blood of these patients on the pathogenesis of Pseudomonas aeruginosa is basically an unexplored area. Rather than using standard laboratory media, we grew P. aeruginosa in whole blood from either healthy volunteers or trauma patients. The specific changes in the P. aeruginosa transcriptome in response to growth in blood from trauma patients reflect the adaptation of this organism to the bloodstream environment. This knowledge is vital for understanding the strategies this pathogen uses to adapt and survive within the host during systemic infection. Such information will help researchers and clinicians to develop new approaches for treatment of sepsis caused by P. aeruginosa in trauma patients, especially in terms of recognizing the effects of specific therapies (e.g., iron, zinc, or mannitol) on the organism. Further, this information can most likely be extrapolated to all patients with P. aeruginosa septicemia.

## INTRODUCTION

Trauma patients (TPs) are at increased risk of infections due to the nature of their injuries. Violation of skin integrity in an invariably contaminated setting allows inoculation of the wound with bacteria. The need for mechanical ventilation, hemodynamic monitoring, and blood transfusions increases the vulnerability of trauma patients to infection. Sepsis occurs in approximately 25% of trauma patients (1, 2). Sepsis has recently been defined as life-threatening organ dysfunction caused by a dysregulated host response to infection (3). In the United States, one million individuals with sepsis are hospitalized annually with a yearly death rate of about 200,000 (4, 5). Septic shock, a state in which the blood pressure drops and cannot be restored by fluid resuscitation and serum lactate levels rise, is much more likely to result in death than sepsis alone (3, 6, 7). It has been estimated that the mortality rate among U.S. patients with septic shock increases by 7.6% for each hour of delay in administration of an effective antimicrobial agent (8, 9). The average annual cost of caring for septic patients reached $20 billion in 2011 (3, 4, 8). Despite advances in modern medicine, including vaccines, antibiotics, and better acute care, if not recognized early and treated promptly, sepsis remains the primary cause of death from infection.

Sepsis in trauma patients is usually accompanied by bloodstream infection and is frequently associated with Gram-negative bacteria, such as Pseudomonas aeruginosa. This opportunistic human pathogen is one of the primary causes of sepsis, with an incidence rate of ∼11% in males and ∼4% in females (10). Currently, P. aeruginosa is resistant to many available antibiotics and is considered one of the serious multidrug-resistant threats in the United States and worldwide (https://cdc.gov/drugresistance/biggest_threats.html; http://who.int/news-room/fact-sheets/detail/antimicrobial-resistance). P. aeruginosa can infect all body tissues and causes both acute and chronic infections. Moreover, it is one of the most common pathogens isolated from hospitalized patients (10). Despite numerous studies, the pathogenesis of P. aeruginosa infection during trauma-induced sepsis is not well understood (11–13).

The trauma patient population is heterogeneous in most aspects, including age, sex, severity and type(s) of injury(ies), recovery time, and clinical outcome (9, 14). Despite this heterogeneity, the host immune response among all types of trauma patients is of a highly similar nature (9, 14, 15). It is characterized by an acute immune response to the presence of damage-associated molecular patterns from traumatized tissues and pathogen-associated molecular patterns from invading microorganisms that stimulate the innate immune system to overexpress inflammatory mediators (9, 14, 16). This uncontrolled immune response leads to the multiple organ dysfunction that occurs in sepsis and to immunosuppression (9, 14, 16).

We recently examined the effect of thermal injury on the expression of P. aeruginosa genes by comparing the level of gene expression when the organism was grown in whole blood from severely burned patients to that of the organisms grown in whole blood from healthy volunteers (HVs) (17). Growth in blood from severely burned patients significantly altered, either positively or negatively, the expression of numerous P. aeruginosa genes, including genes involved in quorum sensing, iron acquisition, and the type III secretion (T3SS) system (17). More importantly, despite the differences among burn patients, including the total surface area of burns and the presence or absence of thermal inhalation injury, the expression level of these genes was comparable in P. aeruginosa grown in blood from all the burn patients tested, parallel to the above-mentioned similarity in the host immune response (17). To further the understanding of P. aeruginosa adaptation during bloodstream infection in trauma patients, and as the trauma patient population is highly heterogenous regarding the sources and type(s) of injury(ies), we examined the changes in P. aeruginosa gene expression in response to its growth in blood from different types of trauma patients with injuries other than burns.

## RESULTS AND DISCUSSION

### Characteristics of the trauma patients used in the study.

We obtained samples of whole blood from 15 subjects, comprised of seven healthy volunteers (HVs) as controls and eight trauma patients (TPs), according to a protocol approved by the Texas Tech University Health Sciences Center (TTUHSC) Institutional Review Board (IRB). The seven HVs, four males and three females aged 24 to 54 years, had no chronic or acute medical conditions. Characteristics of the eight TPs, who presented at University Medical Center, Lubbock, TX, with multisystem traumatic injuries producing injury severity scores (ISS) above 15, are described in Table 1 (2, 18), including the injuries sustained by each patient and antibiotic and blood component therapy administered. Blood samples were obtained from the TPs within 72 h of admission. This time window is appropriate for the study of trauma-induced changes in blood, as the physiological changes that occur transition back to normal within 3 to 8 days after trauma (19).

**TABLE 1:** Clinical characteristics of the trauma patients

ID	Age (yr)	Gender	Transfusion	ISS^*a*^	Injury description^*b*^	Antibiotic(s)
TP3	50	Female	None	16	Fall, acute head injury	Cefazolin
TP7	35	Male	RBC, plasma	26	Major trauma, lacerations of the liver and abdominal wall with a foreign body	Cefazolin/vancomycin/piperacillin
TP10	51	Male	Packed RBC, plasma, platelets	27	MVA, lacerations of liver, spleen, and kidney with large abdominal bleed, large scalp wound	Cefazolin
TP12	24	Female	RBC, plasma, platelets	22	MVA, open-fracture humerus, laceration of diaphragm, traumatic pneumothorax, traumatic brain injury, facial laceration	Cefazolin/gentamicin/penicillin G
TP15	56	Female	Leukoreduced RBC	27	MVA, tibia fracture, closed compression fracture L3 lumbar vertebra	Cefazolin/cefoxitin
TP17	22	Male	None	29	Blunt force trauma, liver laceration extension, mandible fracture, maxillary fracture	Clindamycin
TP18	51	Male	Plasma	38	MVA, closed fracture left humerus, intraparenchymal hemorrhage of brain, open fractures left distal radius/ulna	Cefazolin
TP19	23	Male	None	38	Motorcycle accident, diffuse axonal injury, laceration to right knee, pelvic fracture, bilateral radius fracture	Cefazolin

a Each injury is allocated to one of six body regions (head/neck, face, chest, abdomen/pelvis, extremities, and skin/general) and is assigned an abbreviated injury scale (AIS) score from 1 to 6 (most severe). The AIS scores of the three most severely injured body regions are squared and added together to calculate the injury severity score (ISS) (2, 18).

b MVA, motor vehicle accident.

### Analysis of changes in the P. aeruginosa transcriptome in response to its growth in the blood of TPs.

Initial experiments were done comparing the growth of PA14 in whole blood and Luria-Bertani (LB) broth using a standard growth curve analysis, measuring the optical density at 600 nm (OD_600_) as well as the CFU/ml. Whole blood supported the growth of PA14 at different stages of growth (early log, mid-log, late log, and early stationary) (see Fig. S1A and B in the supplemental material). Based on these results, and to understand the effect of trauma-induced changes within the bloodstream on the expression of the P. aeruginosa transcriptome, we grew the P. aeruginosa strain UCBPP-PA14 (PA14) (20) in whole blood from individual TPs and HVs for 4 h at 37°C. Growth of PA14 was comparable in blood from TPs and HVs, reaching 1.9 × 10^8^ and 1.4 × 10^8^, respectively, at 4 h postinoculation (Fig. S1C). PA14 was harvested from the blood; mRNA was extracted from the pelleted bacteria and used to construct whole transcriptome shotgun sequencing (RNA-seq) libraries that were sequenced using next-generation sequencing technology. The Rockhopper 2 system was used for downstream analysis of the RNA-seq data (21).

10.1128/mSystems.00312-18.1 FIG S1 Comparison of the growth of Pseudomonas aeruginosa strain PA14 in whole blood versus Luria-Bertani broth (LB). Standard growth curve analyses were done as previously described (76), with some variations. One milliliter of PA14 grown overnight in LB was pelleted, washed with LB, resuspended in 1 ml of LB, and subcultured into 7.5 ml LB or whole blood to an OD_600_ of 0.02. At 2-h intervals, samples were harvested for determination of the OD_600_ and the numbers of CFU/ml. (A) Growth index by optical density. Red blood cells were lysed with 10× ammonium chloride lysing reagent (ThermoFisher Scientific, Waltham, MA) prior to measuring OD_600_. (B) Growth index by CFU/ml. Bacterial pellets were washed and resuspended in phosphate-buffered saline to the original volume and serially diluted tenfold, and 10-μl aliquots were spotted on LB agar plates. The number of CFU/ml was determined using the following formula: CFU/ml = (CFU counted × dilution factor) × 100. (C) Comparison of CFU/ml of PA14 grown in the blood of 5 HVs and 5 TPs. Flasks containing 7.5 ml whole blood were inoculated with ∼10^6^ CFU/ml of PA14, similar to the approach described by Mereghetti et al. (77). At 4 h, the cultures were harvested and an aliquot of each was processed as described for panel B to determine CFU/ml. Analysis using the multiple *t* test for significance revealed no significant differences between the two groups; *P =* 0.350339 and 0.393083. Download FIG S1, TIF file, 0.3 MB. Copyright © 2019 Elmassry et al. 2019 Elmassry et al. This content is distributed under the terms of the Creative Commons Attribution 4.0 International license.

**FIG 1:**
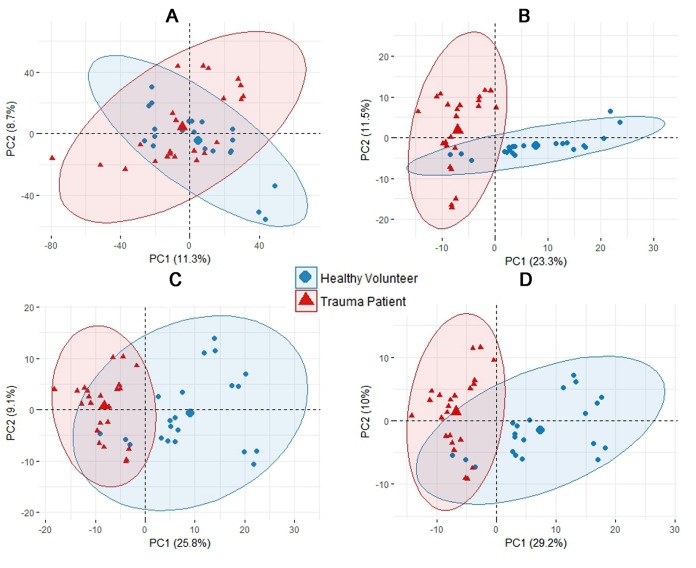
Transcriptomic multivariate analyses for P. aeruginosa PA14 grown in blood from TPs and HVs. Principal component analysis (PCA) plots are shown. (A) Plot of the entire PA14 transcriptome. (B) Differential expression analysis (DEA). (C) Orthogonal partial least square-discriminant analysis (OPLS-DA). (D) Combination of DEA and OPLS-DA.

Initial examination of the entire transcriptomic profiles of the TPs and HVs by principal component analysis (PCA) failed to reveal any notable clustering effects with respect to age, sex, or type of trauma (Fig. 1A) (22). This is most likely due to the inherent biological variability of the subjects (both TPs and HVs) as well as variability originating from the diverse types of trauma sustained by the TPs. It is possible that a larger number of subjects would reveal such clustering. However, we focused on the common effect of trauma on the transcriptome of the pathogen to understand its adaptation in the bloodstream environment. Since such large variability undermines the confidence of traditional statistics (fold change and *P* value), we performed additional analyses. First, we used differential expression analysis (DEA), a commonly used tool for analyzing RNA-seq data, to determine the genes whose expression was up- or downregulated. This analysis showed that of the 5,983 genes in the PA14 transcriptome, 428 were differentially expressed when PA14 grown in whole blood from TPs was compared with PA14 grown in whole blood from HVs (Fig. 1B). We next used a multivariate analysis tool, orthogonal partial least square-discriminant analysis (OPLS-DA) (23), which revealed 461 differentially expressed genes between the two groups (Fig. 1C). These genes were selected according to their variable importance in projection (VIP) scores, corresponding weights, and coefficient values (Fig. S2A and Table S1) (23, 24). The OPLS-DA allowed us to separate the predictive variation from orthogonal variation, enhance interpretation of class variability, and construct a model that can be used in allocation of a new sample to the correct group (24). The OPLS-DA model (Fig. S2B) extracted 20.2% of the total variance and explained 99.1% (*R*^2^ = 0.991) of the variance between PA14 gene expression in blood from HVs and TPs. This model had good predictive power, as indicated by the prediction goodness parameter (*Q*^2^ = 0.677) computed using cross-validation with sensitivity and specificity of 91.67% and 100%, respectively.

10.1128/mSystems.00312-18.2 FIG S2 Changes in the Pseudomonas aeruginosa strain PA14 transcriptome induced by growth in the blood of trauma patients (TPs) compared to its growth in the blood of healthy volunteers (HVs). (A) OPLS-DA score plot that was used to classify the transcriptomic profiles for P. aeruginosa grown in blood from TPs and HVs (23, 24). (B) Dot plot of the PA14 transcriptome based on OPLS-DA. Weights (*x* axis) and VIP scores (*y* axis) of gene expression are shown. The red and green dots represent genes with the highest and lowest significant fold change of expression in blood from TPs, respectively. The OPLS-DA model extracted 20.2% of the total variance and explained 99.1% (*R*^2^ = 0.991) of the variance between PA14 gene expression in blood from HVs and TPs. The prediction goodness parameter, *Q*^2^ = 0.677, was computed using cross-validation with sensitivity and specificity of 91.67% and 100%, respectively. (C) Venn diagram showing the consolidation of the differential expression analysis (DEA) and OPLS-DA. This consolidation revealed 285 genes differentially expressed in PA14 grown in the blood of TPs compared to its growth in the blood of HVs. Download FIG S2, TIF file, 0.7 MB. Copyright © 2019 Elmassry et al. 2019 Elmassry et al. This content is distributed under the terms of the Creative Commons Attribution 4.0 International license.

10.1128/mSystems.00312-18.7 TABLE S1 Selected genes exemplifying significant upregulation, downregulation, or no significant change in expression between PA14 grown in blood from TPs and HVs (23–25). Download Table S1, PDF file, 0.2 MB. Copyright © 2019 Elmassry et al. 2019 Elmassry et al. This content is distributed under the terms of the Creative Commons Attribution 4.0 International license.

To gain even more confidence that we had determined the unique transcriptional changes of PA14 in response to trauma-induced changes in blood, we extracted the genes common to DEA and OPLS-DA by combining the two analyses manually and found 285 genes that were differentially expressed (Fig. S2C and Table S2). Selection criteria for differential expression required genes to have a fold change of ≥1.5 and a *q* value of ≤0.05 to be considered significant, plus a VIP of 1.5 or greater, a weight of ≥0.02 or ≤−0.02, and coefficient of ≥0.001 or ≤−0.001. The *q* value was obtained by adjusting the *P* value using the Benjamini-Hochberg procedure (25). Combining the results from both DEA and OPLS-DA achieved higher explanatory power (39.2%) (Fig. 1D) than results from DEA alone (34.8%) (Fig. 1B) or OPLS-DA alone (34.9%) (Fig. 1C). This innovative stringent analysis selected only those differentially expressed genes that are common among all TPs and excluded any genes with variable expression among the TPs.

10.1128/mSystems.00312-18.8 TABLE S2 Expression levels of the differentially expressed genes (TPs versus HVs) and their product description and subsystem classification (26, 79). Download Table S2, PDF file, 0.2 MB. Copyright © 2019 Elmassry et al. 2019 Elmassry et al. This content is distributed under the terms of the Creative Commons Attribution 4.0 International license.

### Functional analysis of the differentially expressed genes.

**FIG 2:**
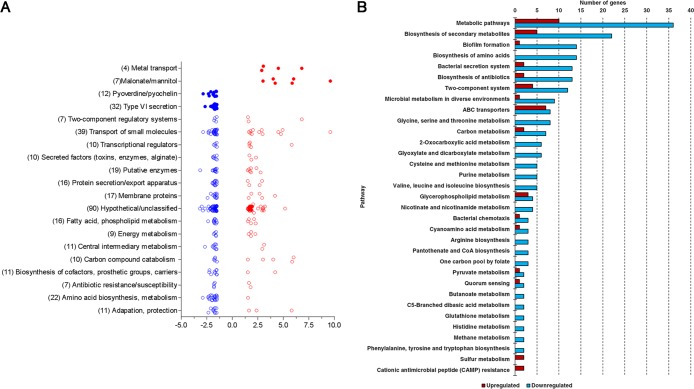
Pathway analyses of the differentially expressed genes. (A) Graph of PseudoCAP function class assignments of the differentially expressed genes and their regulation. PseudoCAP function class assignments (open circles) were graphed if more than 5 genes were differentially expressed in PA14 grown in the blood of TPs compared with PA14 grown in the blood of HVs. Four subclass assignments were made manually for genes of interest (closed circles). (B) Functional analysis of the genes differentially expressed in PA14 grown in the blood of TPs. The graph shows the most frequently represented Kyoto Encyclopedia of Genes and Genome database (KEGG; https://www.genome.jp/kegg/kegg1.html; accessed 10 November 2018) (27, 28) pathways identified for both upregulated (red) and downregulated (blue) genes.

We used three methods of functional analysis to obtain a comprehensive understanding of the classification and function of the 285 differentially expressed genes: Pseudomonas aeruginosa Community Annotation Project (PseudoCAP) function class assignments (http://pseudomonas.com/pseudocap) (26), the Kyoto Encyclopedia of Genes and Genome database (KEGG; https://www.genome.jp/kegg/) (27, 28), and Pathway Tools, version 20.0, software (http://bioinformatics.ai.sri.com/ptools/) (all accessed 10 November 2018) (29, 30). Using PseudoCAP, we found that the PA14 genes differentially expressed in blood from TPs were divided among 23 different functional classes plus an unclassified group, with some genes placed in more than one class (Fig. 2A). Besides the unclassified group (90 genes), functional class assignments with the greatest number of affected genes included 39 for transport of small molecules, 22 for amino acid biosynthesis and metabolism, and 19 for putative enzymes (the top three) (Fig. 2A). Mapping the same genes through the KEGG database, we identified multiple pathways related to the predominantly downregulated genes, including metabolic pathways, biosynthesis of secondary metabolites, biofilm formation, biosynthesis of amino acids, and bacterial secretion systems (the top five) (Fig. 2B). The top five pathways, including upregulated genes, were metabolic pathways, ABC transporters, biosynthesis of secondary metabolites, two-component systems, and glycerophospholipid metabolism (Fig. 2B). While 16 pathways were represented only by downregulated genes, just two pathways were related to only upregulated genes (Fig. 2B). Finally, we used Pathway Tools to visualize the differentially expressed genes and identify genomic hot spots, i.e., clusters of genes with a similar pattern of regulation. Pathway analysis highlighted the malonate utilization operon and putative mannitol transport genes as significantly upregulated and the hemolysin coregulated protein secretion island-I (HSI-I)-encoded type VI secretion system (H1-T6SS) and the pyoverdine biosynthesis operon as significantly downregulated (Fig. 2A and Fig. S3).

10.1128/mSystems.00312-18.3 FIG S3 Heatmap of PA14 genome showing genes that are differentially expressed when the organism is grown in the blood of TPs relative to HVs. The map was prepared using Pathway Tools, version 20.0, software (http://bioinformatics.ai.sri.com/ptools/; accessed 12 November 2018) (29, 30). The relative amount of up- or downregulation of expression is shown by the color block at the top, with red indicating the highest and green the lowest expression. The genes and operons discussed in the text are indicated. Download FIG S3, TIF file, 3.0 MB. Copyright © 2019 Elmassry et al. 2019 Elmassry et al. This content is distributed under the terms of the Creative Commons Attribution 4.0 International license.

**FIG 3:**
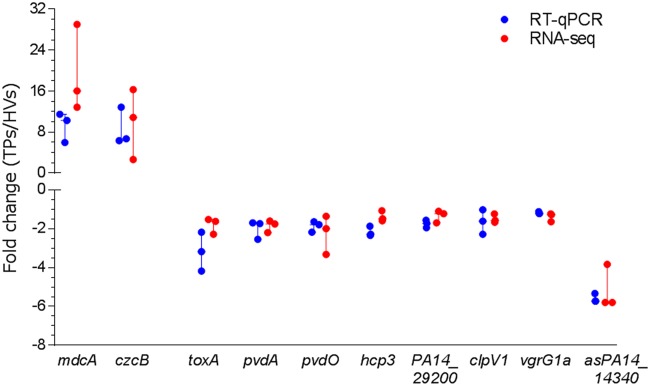
Validation of changes in PA14 gene expression detected in the RNA-seq analysis. Expression of selected genes was determined by RT-qPCR on the same samples used for RNA-seq. Fold change is relative to levels of expression in PA14 grown in whole blood from TPs compared with expression when PA14 was grown in whole blood from HVs. For upregulated genes, *mdcA* represents the malonate utilization operon and *czcB* is a gene for heavy metal efflux. For downregulated genes, *toxA* is an iron-regulated gene; *pvdA*, *pvdL*, and *pvdO* are genes for pyoverdine biosynthesis; *hcp3*, *PA14_29200*, *clpV1*, and *vgrG1* encode proteins related to the T6SS; and *asPA14_14340* is an antisense RNA. For RNA-seq experiments, *q *<* *0.001; for RT-qPCR experiments, *P < *0.05 (*n* = 3).

We selected genes representative of several functional classes and pathways plus one gene carrying an antisense RNA (asRNA) for validation of their changes in expression by quantitative reverse transcription-PCR (RT-qPCR). The relative expression obtained for these two upregulated genes and nine downregulated genes correlated with our RNA-seq results (Fig. 3). The significance of each of these genes will be discussed in detail in the following paragraphs.

### Genes involved in specific metabolic pathways were differentially expressed in response to growth of PA14 in blood from TPs. (i) Genes related to malonate utilization were upregulated.

The growth of PA14 in blood from TPs induced the expression of the genes related to malonate decarboxylation and utilization, which are organized in an operon comprised of seven genes. The first five genes of the operon (*mdcA*, *PA14_02560*, *mdcC*, *mdcD*, and *mdcE*), encoding the alpha, beta, delta, and gamma subunits of the malonate decarboxylase plus the triphosphoribosyl-dephospho-coenzyme A (CoA) synthase (*PA14_02560*), were affected, exhibiting fold changes from 3.0 to 6.0. We validated the differential expression of *mdcA* as the representative gene for the operon by RT-qPCR (Fig. 3). Many bacterial genera, including Acinetobacter baumannii and other *Pseudomonas* spp., such as P. putida, have malonate decarboxylase enzymes that enable them to utilize malonate as a carbon source. Proteins encoded by these genes metabolize malonyl-CoA into acetyl-CoA (31). Recently, Meylan et al. studied the effect of different carbon sources on antibiotic susceptibility (32). Interestingly, the authors found that treating P. aeruginosa with malonate protects it against the lethal effect of the aminoglycoside tobramycin (32). The mechanism of survival was attributed to the inhibition of the tricarboxylic acid cycle, which reduces cellular respiration, thereby impairing tobramycin uptake (32). Similarly, we observed that the MIC of the aminoglycoside kanamycin for PA14 grown in a minimal medium with malonate as a sole carbon source was five times higher than that of PA14 grown in medium with glycerol as a sole carbon source, 1,875 μg/ml and 375 μg/ml, respectively.

**FIG 4:**
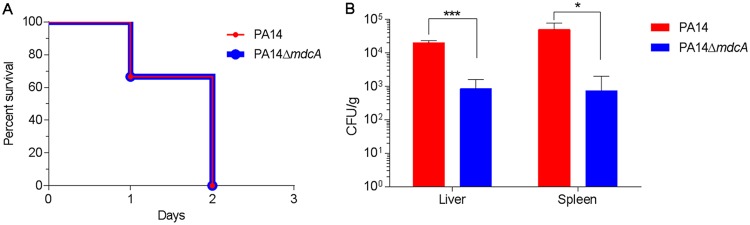
Effect of mutation of *mdcA* on *in vivo* virulence of PA14. (A) The loss of *mdcA* did not affect the survival of thermally injured mice infected with PA14. Adult Swiss Webster mice (three per group) were thermally injured and inoculated with ∼200 CFU of either the PA14 or PA14::*mdcA* strain. The mice were monitored daily for survival after injury/infection. (B) Loss of *mdcA* reduced the systemic spread of PA14. Mice were thermally injured and infected with either the PA14 or PA14::*mdcA* strain as described for panel A. At 24 h after injury/infection, the mice were euthanized and the livers and spleens were obtained, weighed, homogenized in PBS, serially diluted 10-fold, and plated to determine the number of CFU/g of tissue. Values represent the means from three independent experiments ± standard errors of the means. *, *P < *0.05; ***, *P < *0.001.

Considering that malonate is central to carbon metabolism and energy production, these results led us to examine whether the malonate utilization operon is essential for the virulence of P. aeruginosa in the murine model of thermal injury (33). We tested the *in vivo* virulence of PA14 and its PA14/MrT7::*PA14_02550*-662 (PA14::*mdcA*) transposon mutant (34) by inoculating two groups of thermally injured mice (six/group) with approximately 200 CFU of the PA14 or PA14::*mdcA* strain. Although the percent survival of mice infected with the PA14::*mdcA* strain was similar to that of mice infected with PA14 by day two after injury and infection (Fig. 4A), the bacterial burden within the livers and spleens was significantly lower in PA14::*mdcA* mutant-infected mice (Fig. 4B). This suggests that the disruption of malonate utilization in P. aeruginosa negatively influences its fitness in the murine model of thermal injury.

### (ii) Genes related to sugar uptake were upregulated.

The highest level of upregulation was detected with the genes *PA14_34370* (10-fold change) and *PA14_34390* (34-fold change), which encode orthologs to MtlK and MtlG, respectively. Found in P. protegens, P. syringae, and P. fluorescens, these proteins transport polyols (sugar alcohols) such as mannitol and sugars such as maltose. As mannitol has been shown to induce the expression of these transporters in P. fluorescens (35), it was not surprising to find these genes upregulated in PA14 grown in the blood from our TPs, since mannitol is commonly used in the initial treatment of trauma patients to increase plasma osmolality and reduce intracranial pressure (36).

### (iii) Genes related to uptake and metabolism of amino acids were downregulated.

Among the 22 genes in the “amino acid biosynthesis and metabolism” classification, all but one gene were downregulated by 1.5- to 3-fold (Table S2). An additional five genes in the “transport of small molecules” classification with functions related to amino acid transport were downregulated similarly. Within this group of genes were the three-gene operon *ilvIHC*, encoding proteins involved in biosynthesis of the branched-chain amino acids valine, leucine, and isoleucine, and two genes putatively involved in transport of these amino acids, *PA14_15030* and *PA14_64870*; four genes encoding the glutamate and aspartate transport system, *PA14_46910*, *PA14_46920*, *PA14_46930*, and *PA14_46950*, plus a fifth gene, *ansB*, which is involved in glutamate and aspartate metabolism; and six genes related to glycine and serine metabolism, *gcvH2*, *gcvP2*, *glyA2*, *sdaA*, *gcvT2*, and *glyA1* (Table S2). The induction of expression of genes for glutamate and aspartate transport by these amino acids and their amides has been previously described in P. fluorescens and P. putida (37).

### Metabolomic changes in the blood of trauma patients.

**FIG 5:**
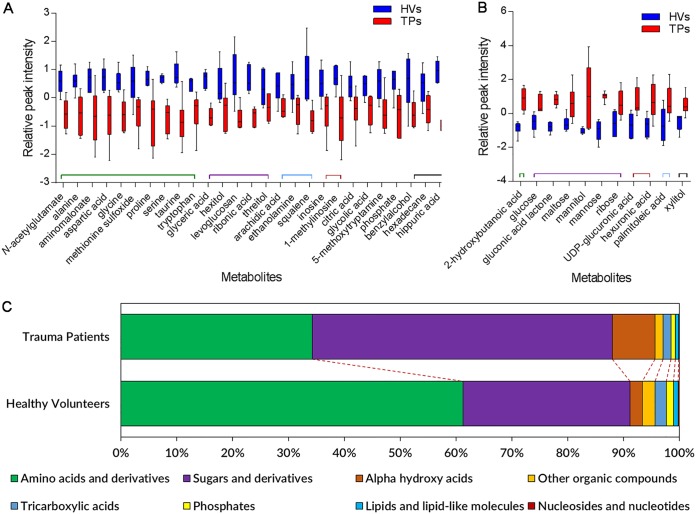
Differential abundance of metabolites in the sera of HVs and TPs. Untargeted metabolomic analysis was done by GC-TOF MS at the West Coast Metabolomics Center (University of California, Davis, CA) on the serum samples collected from six HVs and six TPs. (A) Levels of 27 metabolites were decreased in the serum of TPs (*P < *0.05). Each box plot represents six biological samples, and error bars represent their distribution. (B) Levels of 11 metabolites were increased in the serum of TPs (*P < *0.05). Each box plot represents six biological samples, and error bars represent their distribution. (C) Major metabolite class percentile levels that were significantly different between the serum from the six TPs and six HVs.

We observed numerous transcriptomic changes in multiple pathways related to metabolites involved in primary metabolism, including small molecules, sugars, and amino acids (Fig. 2), in PA14 grown in the blood from TPs and HVs. To understand the role of trauma in inducing metabolomic changes in the blood of these patients, we performed untargeted metabolomic analysis using gas chromatography-time of flight mass spectrometry (GC-TOF MS). This analysis was done at the West Coast Metabolomics Center (University of California, Davis, CA) on the serum samples collected from six HVs and six TPs. Metabolites related to primary metabolism, including carbohydrates and sugar phosphates, amino acids, hydroxyl acids, free fatty acids, purines, pyrimidines, aromatics, and exposome-derived chemicals, were analyzed. Within the serum samples from the 12 subjects, 429 metabolites were detected, of which 135 metabolites were successfully identified. Focusing on those metabolites, PCA showed good separation between the samples from both groups (Fig. S4A). Metabolic clustering analysis confirmed the results from the PCA, producing two branches on the dendrogram based on sample grouping of HVs and TPs (Fig. S4B). Changes in the levels of 38 metabolites were statistically significant (*P < *0.05) between the TPs and HVs and were divided into two groups, one group of 27 whose level was decreased in the serum of TPs (Fig. 5A) and another group of 11 whose level was increased (Fig. 5B). Specific metabolites significantly decreased included ten amino acids and derivatives, including aspartic acid, serine, glycine, and *N-*acetylglutamate (Fig. 5A), while specific metabolites significantly increased included six sugars, including glucose, maltose, mannose, and mannitol (Fig. 5B). These results strongly support the respective decrease and increase in transcription of genes involved in the metabolism of these substrates. Grouping all 135 identified metabolites based on their chemical structure using ClassyFire (https://omictools.com/classyfire-tool; accessed 10 November 2018) (38) showed that the overall levels of sugars and derivatives and alpha hydroxy acids were increased, while amino acids and their derivatives, tricarboxylic acids, lipids, lipid-like molecules, nucleosides and nucleotides, and phosphates, were decreased (Fig. 5C). These results are supported by a previous metabolomics study by Parent et al. that showed that TPs suffer from a decrease in the levels of amino acids in their blood for up to 7 days after trauma, when the levels return to normal (39).

10.1128/mSystems.00312-18.4 FIG S4 Metabolomic analysis of the serum of TPs and HVs. (A) Principal component analysis of the serum sample metabolome from HVs and TPs. (B) Heatmap and hierarchical clustering analysis of the serum sample metabolome from HVs and TPs. For the multivariate statistical analysis of the metabolite data by PCA and clustering analysis, the preprocessed and normalized data set was imported into the ClustVis web tool (https://biit.cs.ut.ee/clustvis/; accessed 20 November 2018) and further analyzed (73). Download FIG S4, TIF file, 0.5 MB. Copyright © 2019 Elmassry et al. 2019 Elmassry et al. This content is distributed under the terms of the Creative Commons Attribution 4.0 International license.

**FIG 6:**
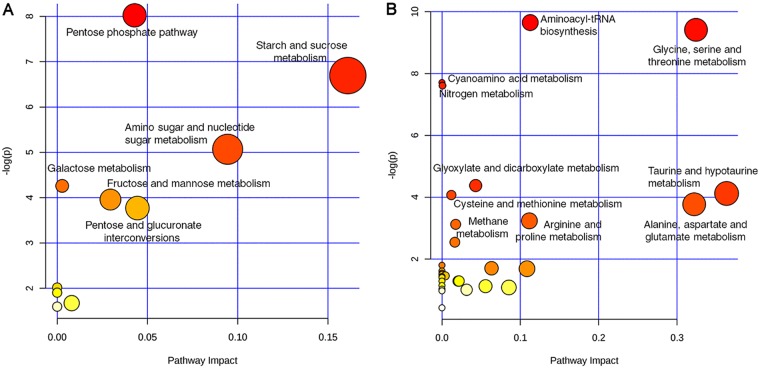
KEGG pathway analysis of the significantly different metabolites. (A) KEGG pathway analysis of the significantly increased metabolites in the serum of TPs. (B) KEGG pathway analysis of the significantly decreased metabolites in the serum of TPs. Labeled pathways in panels A and B are the significantly enriched ones based on statistical significance (*P < *0.05).

To relate the physiological changes in blood metabolites following trauma to specific metabolic pathways, we used MetaboAnalyst (http://www.metaboanalyst.ca/; accessed 10 November 2018) (40) to map the identified metabolites to their corresponding physiologic pathways. Pathway enrichment analysis for the significantly increased metabolites showed the influence on six metabolic pathways, including the pentose phosphate pathway and pathways for starch and sucrose metabolism and amino sugar and nucleotide sugar metabolism (Fig. 6A). In contrast, the significantly reduced metabolites were mapped to ten pathways, including several amino acid metabolism pathways for glycine, serine, and threonine; taurine and hypotaurine; cysteine and methionine; and alanine, aspartate, and glutamate (Fig. 6B). To further integrate our transcriptomic and metabolomic data, we performed genome-scale metabolic model analysis. We used MetExplore (41) to map our omics data to a model of the PA14 metabolic network available in MetaCyc, obtained through the BioCyc database collection (29, 42). Only 73 of the differentially expressed genes and 21 of the metabolites identified in the blood of TPs mapped to the metabolic network. Most noticeably, the effect of trauma-induced changes in blood on the metabolic flux of PA14 is related to amino acid metabolism, specifically glycine, serine, and l-aspartate (Fig. S5).

10.1128/mSystems.00312-18.5 FIG S5 Genome-scale metabolic model analysis mapping the omics data to a model of the PA14 metabolic network. The figure was prepared using MetExplore (41, 78). After mapping the genes and metabolites to the model, enrichment analysis of the metabolic pathways resulted in the enrichment of the following pathways with a *P* value of <0.05: glycine betaine degradation, formyl-THF biosynthesis, folate polyglutamylation, folate transformations, glycine biosynthesis, and l-serine degradation. Reactions related to those pathways were further filtered to include only reactions that have mapped genes and metabolites. This refinement step resulted in finding only 2 reactions occurring that are involved in the previously mentioned pathways. Those reactions included l-serine ammonia-lyase and glycine hydroxymethyltransferase, which are shown as 4.3.1.17-RXN and GLYOHMETRANS-RXN, respectively. Genes mapped to those reactions were *PA14_60890*, *PA14_71460*, *PA14_33010*, *PA14_33030*, and *PA14_71060*, and metabolites mapped to those reactions related to serine and glycine. Download FIG S5, TIF file, 0.4 MB. Copyright © 2019 Elmassry et al. 2019 Elmassry et al. This content is distributed under the terms of the Creative Commons Attribution 4.0 International license.

### Genes involved in virulence were differentially expressed in response to growth of PA14 in blood from TPs. (i) Genes of the T6SS were repressed.

**FIG 7:**
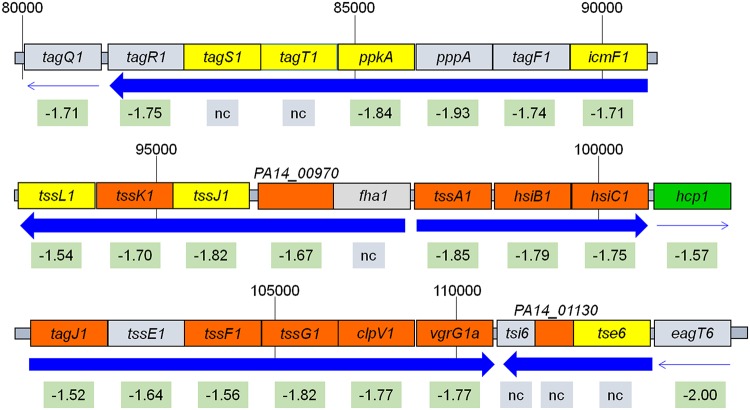
Diagram of the H1-T6SS locus on the PA14 chromosome (not drawn to scale). The fold change in gene expression in the blood of TPs compared to that of HVs is indicated below each gene; nc, no change. Direction of gene transcription is indicated by blue arrows. Thick arrows indicate the genes are transcribed as an operon. Location of the proteins encoded by the genes is indicated by the color of the block: gray, unknown; yellow, cytoplasmic membrane; orange, cytoplasmic; green, extracellular.

We identified over 30 T6SS genes that were downregulated by growth of PA14 in blood from TPs (Table S1) and validated the downregulation of four of these genes (Fig. 3). Found in 25% of Gram-negative bacteria, the T6SS is often encoded by three loci, HSI-I, HSI-II, and HSI-III, plus additional genes scattered over the genome in clusters and pairs (43, 44). While the exact composition of the HSIs varies from species to species, the T6SS functions similarly in all, transporting lethal effector proteins directly into the targeted prokaryotic or eukaryotic cells (44, 45). The majority of the downregulated T6SS genes encode H1-T6SS proteins, with 21 of 27 genes on HSI-I downregulated by 1.5- to 2-fold, including genes encoding eukaryotic (*hcp1*, *vgrG1a*, and *icmF1*) and prokaryotic (*tse1*) effector proteins, as well as structural components of the T6SS secretion apparatus, such as *tssL1*, *tssK1*, *tssE1*, and others (Fig. 7). Besides H1-T6SS, two genes encoding the effector proteins of H3-T6SS (*hcp3* and *hsiC3*) were also downregulated, although no change was observed in the other 15 genes found on the island (Table S2). Additionally, scattered genes encoding prokaryotic effector proteins (Tse3, Tse1-like, and Tse2-like proteins) and the Tsi1-like and Tsi2-like immunity proteins were also downregulated at a similar level (Table S2). Interestingly, none of the 20 genes in the H2-T6SS were affected by growth of PA14 in the blood from TPs.

Enhanced expression of T6SS genes in P. aeruginosa isolates obtained from chronic lung infection of cystic fibrosis patients suggests a role for the T6SS in these infections. Since chronic lung infections of cystic fibrosis patients are polymicrobial, the antimicrobial activity of T6SS may provide P. aeruginosa a fitness strategy during these infections (46, 47). In the setting of bloodstream infection, where there are no competing bacteria, downregulation of the *tse* and *tsi* genes makes sense. Additionally, downregulation of the eukaryotic antipathogenic activity of the H1-T6SS effector orthologs for the Salmonella enterica Sci proteins (encoded by *tagJ1* and *tssJ1*) may play a role in increasing P. aeruginosa virulence during bacteremia (44). The three loci are differentially regulated by quorum-sensing genes, with HSI-I suppressed by both *lasR* and *mvfR*, while HSI-II and -III are positively regulated by these genes (48). Since we did not detect a significant change in the expression of either *lasR* or *mvfR*, trauma is less likely to regulate the H1-T6SS genes through them. Rather, trauma may regulate the H1-T6SS genes through the global regulator RetS, which induces the expression of T3SS genes but represses the expression of HSI-I genes (44, 47). However, there was no significant change in *retS* expression in the blood of TPs compared with that of HVs. At this time, the exact mechanism through which the trauma-related downregulation of the T6SS genes occurs is not known.

### (ii) Genes for heavy metal efflux were upregulated.

Genes of the *czc* cluster, encoding orthologs of the two-component response regulators CzcS and CzcR and the tripartite RND efflux pump CzcCBA, which expels cadmium, zinc, and cobalt, were upregulated between 4.5- and 6.8-fold in PA14 grown in the blood of TPs (Tables S2 and S3 and Fig. S6A) (49, 50). We recovered two transcripts for the putative *czcA*, *PA14_32015* (1,029 nucleotides [nt]) and *PA14_32025* (2,115 nt), rather than one, as found in P. aeruginosa strain PAO1 (49) (Fig. S6A). Analysis of the two *czcA* transcripts using BLASTN 2.8.1 (51) revealed that they matched nt 1 to 1031 and nt 1041 to 3156 of PAO1 *czcA* with 98% and 99% identity, respectively. Similarly, using BLASTX 2.8.1 (52), the translated nucleotide sequences of the transcripts were found to be 99% identical to amino acid (aa) 1 to 323 and aa 348 to 1051 of PAO1 CzcA. We manually assembled *PA14_32015* and *PA14_32025* into a single *czcA* gene (Fig. S6B and C) and interrogated P. aeruginosa PAO1 proteins in the nonredundant proteins database with the translated 3,156-nt sequence. CzcA was 97% identical and 98% similar to P. aeruginosa CzcA and CusA proteins. However, examination of the amino acids sequence revealed the presence of a premature stop codon following aa 342 of the putative PA14 CzcA (Fig. S6D). Whether proteins are translated from either or both transcripts and whether such proteins would be capable of functioning with CzcB and/or CzcC in PA14 has yet to be determined.

10.1128/mSystems.00312-18.6 FIG S6 Analysis of the transcripts *PA14_32015* and *PA14_32025*, which appear to be related to the gene *czcA* in PAO1. (A) The chromosomal arrangements of the *czc* gene cluster in PA14 compared to that in PAO1. Fold enhancement in transcription of the *czc* genes is shown. (B) Since *PA14_32015* and *PA14_32025* matched the nucleotide sequences for these genes in PA14 with 100% identity and shared 98% and 99% identity with PAO1 *czcA*, respectively, manual compilation of these sequences into one DNA sequence resulted in a sequence of 3,156 nt (*czcA*). The intergenic region showing the overlap of the two transcripts is shown. (C) Query of the PAO1 complete genomes (taxid 208964) with *czcA* using BLASTN 2.8.1 (51) returned four matches, including one for the PAO1 complete genome (AE004091.2), which showed an E value of 0.0, and 99% identity (3,212/3,158) with 4 gaps. The gaps (blue highlight) plus 10 transitions and transversions (yellow highlight) were found between nt 921 and nt 1039 ahead of and just past the junction of *PA14_32015* and *PA14_32025* (TGA and GTG, respectively, in red). (D) Interrogation of the translated *czcA* nucleotide sequence against P. aeruginosa PAO1 (taxid 208964) proteins in the nonredundant proteins database using BLASTX 2.8.1 (52) showed that CzcA shares 97% identity with CzcA from multiple strains of PAO1. Examination of these amino acid sequences showed that the amino termini of the two proteins (aa 1 to 306) are identical. Beginning with aa 307 (coinciding with nt 919 to 921, dotted underline in panel C), the changes in the nucleotide sequence produced profound alteration within the next 40 aa of the potential PA14 CzcA protein: 19/40 nonidentical (47.5%), 16/40 identical (40%), and 5/40 similar (12.5%). The most significant of these changes is the premature stop codon generated at aa 343 of the PAO1 CzcA protein. By aa 347 of the PAO1 sequence, the two amino acid sequences are once again identical throughout the carboxy termini of the two sequences except for aa 453, which was similar (identity, 704/705). Download FIG S6, TIF file, 0.2 MB. Copyright © 2019 Elmassry et al. 2019 Elmassry et al. This content is distributed under the terms of the Creative Commons Attribution 4.0 International license.

Independently of *czcCBA*, CzcR in PAO1 negatively regulates *oprD* expression, which leads to carbapenem resistance in P. aeruginosa (53). The upregulation of the putative PA14 *czcR* (*PA14_31960*) may have affected expression of *oprD*, which was downregulated in the blood of TPs by 1.6-fold. *PA14_16660*, which encodes an ortholog of the CadA P-type ATPase transporter involved in the transport of cadmium and zinc in P. putida (54), was upregulated by almost 3-fold. In P. putida, the CadA and CzcCBA transporters overlap in function to eliminate toxic levels of cadmium, zinc, and lead (55). Expression of *czcRS*, *czcCAB*, and *cadA* in P. aeruginosa and P. putida is induced by zinc (50, 53, 55). Many TPs are supplemented with trace metals, including zinc, during their hospitalization, which may explain the increase in transcription of these transporters (56). The unintended increase in CzcR with the concomitant decrease in OprD could lead to failure of carbapenem therapy should such patients develop P. aeruginosa infection (50, 53, 57).

### (iii) Genes for siderophore biosynthesis and transport were downregulated.

**FIG 8:**
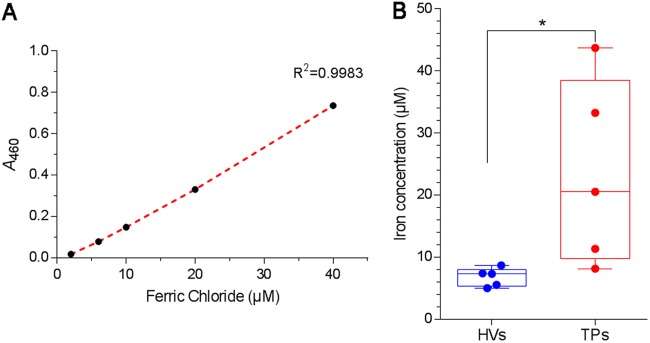
Iron concentration is higher in the serum of TPs than that of HVs. The level of iron in the sera from TPs and HVs was assessed indirectly through its binding to pyoverdine-enriched supernatant (PES) from PA14 grown in the iron-depleted medium Trypticase soy broth dialysate supplemented with 1% glycerol and 50 mM monosodium glutamate. (A) Standard curve was prepared by adding serial dilutions of FeCl_3_ to PES, incubating at room temperature for 10 min, and measuring the absorbance at 460 nm. (B) Iron concentration in serum of HVs and TPs was determined by adding 100 μl of each serum to be tested to 1 ml of PES, incubating as described for panel A, obtaining the *A*_460_, and calculating the equivalent amount of iron from the standard curve. The boxes represent the median from five independent experiments, and upper and lower whiskers represent the range of distribution. *, *P < *0.05.

P. aeruginosa synthesizes two types of small iron-chelating compounds, known as siderophores, for iron acquisition when iron is scarce in the environment (58, 59). Nine genes related to the biosynthesis and transport of the high-affinity pyoverdine siderophore were significantly downregulated in PA14 grown in blood from TPs from 1.6- to 2.3-fold. We validated the differential expression of three of these genes, *pvdA*, *pvdL*, and *pvdO* (Fig. 3). The pyoverdine biosynthetic genes are known to be downregulated in the presence of iron through PvdS (60). Not only was *pvdS* expression downregulated by 1.6-fold, another iron-regulated gene also regulated by PvdS, *toxA* (the gene encoding exotoxin A), was downregulated at a level similar to that of the pyoverdine genes (Fig. 3) (60). The gene encoding HasR hemophore receptor was also significantly downregulated by 2.3-fold. Three genes related to the synthesis and uptake of the low-affinity pyochelin siderophore were also downregulated (Table S2). A simple explanation for these findings is that the level of iron in the blood of TPs is higher than that in the blood of HVs. We isolated the serum fraction from the blood of TPs and HVs and assessed the level of iron in the serum indirectly through its binding to pyoverdine (61, 62). The levels of iron in the blood of the TPs were significantly higher (*P* < 0.05) than those in the blood of HVs (Fig. 8).

### Growth in blood from TPs significantly alters the expression of asRNA transcripts in PA14.

Over the past several years, transcriptomic studies of various bacteria, such as P. aeruginosa, Escherichia coli, Bacillus subtilis, and Helicobacter pylori, have reported the presence of widespread transcriptional activity that does not result in mRNA but in asRNA and small regulatory RNAs (63–66). Using the data from the strand-specific RNA-seq analysis, we compared the expression of the asRNA transcripts in PA14 grown in blood from TPs and HVs and found 68 differentially expressed asRNA transcripts, three of which were highly downregulated (Table S3). An asRNA to *PA14_14340*, which encodes an *S*-adenosyl-l-methionine-dependent methyltransferase, was downregulated by 54-fold (Table S3), which we confirmed by RT-qPCR (Fig. 3). An asRNA to *PA14_39520*, which encodes a putative hydroxylase subunit, was downregulated by 22-fold, while an asRNA to the *pepP* gene, encoding the PepP aminopeptidase, was downregulated by 18-fold (Table S3). However, no change in expression of the sense transcripts for any of these three genes was observed. Interestingly, we detected full-length asRNA transcripts for all four 16S rRNA genes and all four 23S rRNA genes that were upregulated by 4-fold and 6.6-fold, respectively. As rRNA transcripts were removed during the preparation of the cDNA libraries, we do not know if these asRNAs had any effect on the expression of the 16S or 23S rRNA genes.

10.1128/mSystems.00312-18.9 TABLE S3 Predicted antisense RNAs and other RNA products differentially expressed in PA14 grown in blood from TPs compared to its growth in blood from HVs (63). Download Table S3, PDF file, 0.3 MB. Copyright © 2019 Elmassry et al. 2019 Elmassry et al. This content is distributed under the terms of the Creative Commons Attribution 4.0 International license.

Similar to the results obtained by Eckweiler and Häussler (63), when asRNA transcripts were detected for genes whose expression was altered by growth in blood from TPs, there was little correlation to the expression of the mRNA of those genes: asRNA for *PA14_21570* was upregulated by 6-fold, while sense transcription of this gene was upregulated by 3-fold; similarly, the asRNA for *PA14_32250* was upregulated 4.3-fold while the mRNA was upregulated by 5-fold, and the asRNA for *PA14_33870* was enhanced by 2.3-fold and mRNA by 1.8-fold (Table S3). In only one case did the asRNA and mRNA transcription diverge: the asRNA for *leuA* was upregulated by 3.6-fold and transcription of *leuA* message was downregulated by 2.1-fold (Table S3). We also detected a number of upregulated asRNA transcripts in common with those of Eckweiler and Häussler (63): asRNAs to *bfrA* (2.2-fold), *leuA* (3.6-fold), *mvfR* (3-fold), and *PA14_45470*, which codes for a putative glutathione *S*-transferase (2-fold) (Table S3). At this time, the role of these 68 asRNAs has yet to be determined.

### Comparison of the transcriptomic changes induced in P. aeruginosa by blood from trauma patients, burn patients, and thermally injured mice.

The burn patient population is considered a small subset of the trauma patient population, although it is more homogeneous than the trauma patient population. Therefore, we compared results from our current study with those from our recently published study on the transcriptomic changes in PA14 grown in blood from severely burned patients (17). When we reanalyzed the previously published data using the same system of analysis as that used in this study, we found that 100 genes were differentially expressed in both studies, with 59 genes similarly regulated (either both up or both down), while 41 genes were oppositely regulated (Table S4). Among the genes that were similarly regulated, we found that six genes of the pyoverdine biosynthetic pathway plus the gene encoding the ferripyoveridine receptor were downregulated in both studies; similarly, three genes involved in the uptake and metabolism of amino acids were downregulated (Table 2 and Table S4). More interestingly, the five genes related to malonate utilization were upregulated in both studies (Table 2 and Table S4). As deletion of *mdcA*, the first gene in the malonate utilization operon, reduced systemic spread of PA14 within thermally injured mice (Fig. 4B), we compared the expression of these five genes (*mdcA*, *PA14_02560*, *mdcC*, *mdcD*, and *mdcE*) in PA14 grown in blood from thermally injured mice to that in blood from uninjured mice using RT-qPCR. We found that four of the genes were significantly upregulated (*P < *0.01) (Table 3). The fact that the malonate utilization operon was upregulated under all three conditions underlines the importance of malonate utilization by P. aeruginosa during systemic infection. Further study will be needed to determine the exact role played by malonate utilization in the pathogenesis of P. aeruginosa.

**TABLE 2:** Selected PA14 genes differentially expressed in blood from SBPs and TPs

Locus	Gene^*a*^	Product	Fold change^*b*^	
			SBP	TP
PA14_02570	*mdcC*	Malonate decarboxylase subunit delta	**11.63**	**6.00**
PA14_02550	*mdcA*	Malonate decarboxylase subunit alpha	**12.13**	**5.86**
PA14_02560	*PA14_02560*	Triphosphoribosyl-dephospho-CoA synthase	**20.73**	**4.20**
PA14_02590	*mdcE*	Malonate decarboxylase subunit gamma	**8.54**	**4.00**
PA14_02580	*mdcD*	Malonate decarboxylase subunit beta	**13.17**	**3.00**
PA14_33500	*pvdH*	Diaminobutyrate–2-oxoglutarate aminotransferase	*−4.61*	*−2.35*
PA14_33680	*fpvA*	Ferripyoverdine receptor	*−4.34*	*−1.56*
PA14_33690	*pvdE*	Pyoverdine biosynthesis protein PvdE	*−2.36*	*−2.11*
PA14_33700	*pvdF*	Pyoverdine synthetase F	*−4.94*	*−1.89*
PA14_33710	*pvdO*	Protein PvdO	*−4.71*	*−1.83*
PA14_33720	*pvdN*	Protein PvdN	*−2.45*	*−1.72*
PA14_33810	*pvdA*	l-Ornithine N5-oxygenase	*−3.81*	*−2.27*
PA14_46970	*ansB*	Glutaminase-asparaginase	*−5.99*	*−2.29*
PA14_62130	*ilvC*	Ketol-acid reductoisomerase	*−2.77*	*−1.54*
PA14_62150	*ilvH*	Acetolactate synthase 3 regulatory subunit	*−2.35*	*−1.70*

a Genes specifically discussed in the text; for the complete list of 100 genes, see Table S4.

b Upregulation (boldface) and downregulation (italics) of gene expression in PA14 grown in blood from severely burned patients (SBPs) or TPs compared to levels for healthy volunteers.

**TABLE 3:** Differentially expressed genes related to malonate utilization^*a*^

Locus	Gene	Product	Fold change^*a*^		
			TP	SBP	TIM
PA14_02550	*mdcA*	Malonate decarboxylase subunit alpha	5.9^*b*^	12.1^*b*^	3.1^*c*^
PA14_02560	*PA14_02560*	Triphosphoribosyl-dephospho-CoA synthase	4.2	20.7	3.1
PA14_02570	*mdcC*	Malonate decarboxylase subunit delta	6.0	11.6	3.3
PA14_02580	*mdcD*	Malonate decarboxylase subunit beta	3.0	13.2	3.23

a PA14 was grown in blood from TPs, SBPs, or thermally injured mice (TIM) and compared with PA14 grown in blood from HVs or healthy mice.

b Values obtained by RNA-seq, *q *<* *0.01.

c Values obtained by RT-qPCR, *P *<* *0.01 (*n* = 3).

10.1128/mSystems.00312-18.10 TABLE S4 PA14 genes differentially expressed in blood from both severely burned patients (SBPs) (17) and TPs. Download Table S4, PDF file, 0.05 MB. Copyright © 2019 Elmassry et al. 2019 Elmassry et al. This content is distributed under the terms of the Creative Commons Attribution 4.0 International license.

Cornforth et al. recently examined the transcriptomes of P. aeruginosa present in human infections, specifically CF sputum and infections of chronic wounds and burns (67). These types of infections are frequently associated with biofilm formation and result in long-term treatment of the patients with multiple antibiotics. Analysis of gene expression in the different P. aeruginosa transcriptomes was related to human versus *in vitro* samples and revealed that transcriptomes from these two types of samples are distinct (67). Overall, they reported that differential expression of genes related to metabolism was higher in *in vitro* samples, as was that of quorum-sensing genes, while genes determining antibiotic tolerance were expressed at higher levels in human infections (67). Additionally, a core group of P. aeruginosa genes that distinguish human infection from *in vitro* transcriptomes was defined (67).

In our current study, we found genes related to amino acid metabolism, iron acquisition, and type VI secretion to be downregulated in the blood of TPs, while genes for malonate utilization and mannitol uptake were upregulated. Most quorum-sensing genes, genes related to antibiotic tolerance, and the core group of genes that distinguish the human infection transcriptome were unaffected. Our study differs in four areas: (i) we used human blood as an *ex vivo* medium for growth of a laboratory strain of P. aeruginosa; (ii) rather than chronic infection, where biofilms are common, our model is of acute septicemia and examined adjustment of PA14 to planktonic growth within 4 h; (iii) we compared the effect of trauma-induced changes in the blood on the PA14 transcriptome to that when grown in blood from noninjured healthy individuals rather than to growth in a laboratory medium; and (iv) the exposure of PA14 to antibiotics was limited to any residual drug in the patients’ blood at the time of the blood draw. All eight of the TPs received prophylactic antibiotic coverage upon admission (Table 1), but only TP12 received an antipseudomonal antibiotic (gentamicin), and that was 28 h prior to the blood draw. Recently, Murray et al. showed that exposure of P. aeruginosa PA14 to sublethal concentrations (1/2 the MIC) of various antibiotics significantly altered expression of many of its genes with exposure to the antipseudomonal third-generation cephalosporin cefoperazone, resulting in upregulation of 24 genes (68). Based on the half-life of the different antibiotics administered, only TP17 and TP24, who received cefazolin prior to their blood draws (at 3 h 4 min and 27 min, respectively), had antibiotic levels higher than 12.5% of the dose. Despite the lack of effect seen on most genes related to antibiotic tolerance, we did observe downregulation of *oprD*, likely related to an increase in *czcR*, which is associated with carbapenem resistance, and a 5-fold increase in kanamycin MIC related to an increase in malonate utilization. This suggests that changes in metabolites within the blood of TPs secondarily lead to antibiotic tolerance prior to administration of the antibiotic.

### Conclusions.

Utilizing our unique approach of growing P. aeruginosa in blood from TPs, we were able to study the impact of trauma-induced changes in blood on the P. aeruginosa transcriptome. Our study provides strong evidence that P. aeruginosa adapts to this unique trauma-induced environment during bloodstream infection of TPs. The observed upregulation of genes related to maltose/mannitol uptake and heavy metal efflux suggests that during systemic infection in TPs, P. aeruginosa adapts to the presence of therapeutic molecules in the bloodstream of these patients, such as mannitol and zinc. The downregulation of siderophore biosynthesis and uptake in the iron-rich environment found in the bloodstream of TPs shows further adaptation to environmental conditions, as does the downregulation of amino acid biosynthesis and transport systems. Finally, downregulation of the H1-T6SS as well as genes for additional effector/immunity proteins used to compete against other bacteria shows the ability of P. aeruginosa to adjust its virulence to the situation, increasing its virulence to host macrophages while conserving its response in an environment lacking competition. Future work will be directed to the study of the metabolomic changes in blood of TPs infected with P. aeruginosa and any specific factors responsible for the observed changes in the P. aeruginosa transcriptome. In terms of administering different treatments to the trauma patient, such as iron, zinc, and mannitol, knowledge of the changes in gene expression related to bacterial transport and utilization of these products may impact therapy for these patients.

## MATERIALS AND METHODS

### Ethics statement for collection of human blood.

This study was approved by the TTUHSC Institutional Review Board, IRB protocol L15-057. Informed written consent was obtained upon the patient’s admission to University Medical Center, Lubbock, TX, by a staff member of the Clinical Research Institute (CRI) at TTUHSC in compliance with ethical practices. If the patient was unable to provide such consent, informed written consent was obtained from designated next of kin by a CRI staff member according to the protocol approved by the IRB. Informed written consent was obtained from healthy volunteers by an individual with appropriate training mandated by the IRB under this IRB-approved protocol. No children were involved in this study. Subjects were required to be between 18 and 89 years of age. Patients admitted to the study had multiple traumatic injuries resulting in an injury severity score above 15 for each patient (TPs) (Table 1) (2, 18). Healthy volunteers (HVs) with any acute or chronic medical conditions were excluded.

CRI staff obtained the blood samples from the TPs and HVs by venipuncture according to the protocol approved by the IRB. Blood was collected from the TPs within 72 h of their admission to the hospital. A total of 25 ml of blood was collected from each person into four BD Vacutainer tubes (Becton Dickinson, Franklin Lakes, NJ), one for separating the serum and three containing 0.35% sodium polyanetholesulfonate as an anticoagulant in 1.7 ml of 0.85% sodium chloride (SPS tubes) for PA14 growth. Per the IRB protocol, blood samples were deidentified (names and HIPAA identifiers removed), and the samples were given unique numbers by the CRI staff before the samples were sent to the research laboratory. All methods performed on the samples were in compliance with the relevant guidelines and regulations of the IRB-approved protocol.

### Bacterial strains, media, and growth conditions.

P. aeruginosa strain UCBPP-PA14, a prototrophic strain of P. aeruginosa originally isolated from a wound infection, was used in all experiments (20). It is a highly virulent strain that causes disease in a wide range of organisms. An isogenic mutant of PA14, the PA14/MrT7::*PA14_02550*-662 strain, carrying the Mariner transposon *MAR2Tx7* inserted at nucleotide 662 in *mdcA* (PA14::*mdcA*) (34), was used to examine the importance of the malonate utilization operon in virulence. Strains were routinely grown overnight and maintained at 37°C in LB broth prior to subculture into whole blood. Gentamicin was added at 50 µg/ml to maintain the transposon in the PA14::*mdcA* mutant.

### Growth of P. aeruginosa strains in whole blood.

Blood from the three SPS tubes was pooled, and 7.5-ml aliquots were added to each of three separate 25-ml flasks (three independent replicate experiments). To avoid excessive red blood cell (RBC) lysis, the blood was used immediately after collection. We inoculated each flask with 10^6^ CFU/ml of overnight LB broth culture of the PA14 or PA14::*mdcA* strain. The inoculated blood cultures were incubated in a 37°C shaking water bath. At 4 h postinoculation and when bacterial growth reached about 10^8^ CFU/ml, the cultures were processed.

Blood cultures were diluted 1:1 with phosphate-buffered saline (PBS) and gently layered over lymphocyte separation medium (Lonza, Walkersville, MD), a mixture of Ficoll and sodium diatrizoate (Hypaque) with density adjusted to 1.077 g/ml, at a blood/medium ratio of 3:2 (9 ml blood to 6 ml medium). The layered tubes were centrifuged at 400 × *g* for 15 min to separate the blood into layers. The upper and middle layers containing the lymphocytes and granulocytes, respectively, were discarded. The bottom layer, containing RBC and bacteria, was suspended in 1.9 ml of buffer EL (erythrocyte lysis buffer) (Qiagen, Valencia, CA), and the samples were centrifuged at 8,000 rpm for 4 min. The supernatant containing lysed RBC was discarded; the pellet was resuspended in 1.9 ml of buffer EL and centrifuged again. The process was repeated until all RBCs were lysed and only bacterial cells remained in the pellet. The bacterial cells were resuspended in 3 ml of LB broth and 6 ml of RNAprotect (Qiagen) and incubated for 5 min at room temperature. Cells were again pelleted and stored at −80°C until processing.

### Bacterial RNA extraction.

Bacterial pellets were lysed by the addition of lysozyme and proteinase K for 15 min at room temperature. RNA was extracted using the RNeasy minikit (Qiagen) according to the manufacturer’s recommendations, and the RNA solution was digested with the RNase-free DNase set (Qiagen), followed by on-column DNase digestion to eliminate any remaining traces of genomic DNA. The purified RNA was quantified using a NanoDrop spectrophotometer (NanoDrop Technologies, Wilmington, DE), and the integrity of the RNA was assessed using RNA Nano Chip on an Agilent 2100 Bioanalyzer (Agilent, Palo Alto, CA). The integrity of RNA was further measured on a TapeStation 2200 (Agilent) by following the manufacturer’s instructions. RNA samples with 1.8 to 2.2 ratio of absorbance at 260/280 nm were kept for further analysis. Only samples with an RNA integration number (RIN) greater than 8.0 were used for cDNA library preparation.

### Construction of the RNA-seq library and sequencing of the cDNA libraries.

rRNA was depleted from total RNA with the Ribo-Zero rRNA removal kit for bacteria (Epicentre Biotechnologies, Madison, WI). Enriched mRNA samples were run on the TapeStation 2200 to confirm reduction of 16S and 23S rRNA. RNA-seq libraries were constructed from ∼1 μg of rRNA-depleted mRNA using the TruSeq stranded mRNA library preparation kit by following the manufacturer’s protocol (Illumina, San Diego, CA). RNA was fragmented using divalent cations under elevated temperature and primed for cDNA synthesis at 94°C to obtain a median insert size of 200 bp per fragment. The fragmented RNA templates were primed with random hexamers, and the first strand was synthesized by four cycles of 25°C for 10 min, 42°C for 50 min, and 70°C for 15 min. Following second-strand synthesis (16°C for 1 h), end repair was performed to generate blunt ends followed by adenylation of the 3′ blunt-ended cDNAs to allow for subsequent ligation of multiple indexing adaptors and hybridization onto the flow cell. cDNA fragments were enriched using 15 PCR cycles of 98°C for 10 s, 60°C for 50 s, and 72°C for 15 s. The libraries were quantified using a Qubit fluorometer (Life Technologies, Carlsbad, CA), and the quality was analyzed with the TapeStation 2200 using the D1K tape for validating the insert size and purity. The multiplexed cDNA libraries were sequenced using a MiSeq Sequencer (Illumina) using V3 chemistry by following the manufacturer’s protocol. Paired-end sequencing was performed to obtain 75-bp reads using a 150-cycle reagent cartridge.

### Analysis of the RNA-seq data.

The sequencing data of each sample have been deposited under BioProject accession number PRJNA436010 in the National Center for Biotechnology Information (NCBI) BioProject database (accessed 11 November 2018). The Rockhopper 2 system (21) was used in RNA-seq data analysis, implementing reference-based transcript assembly with UCBPP-PA14 as a reference genome (http://www.pseudomonas.com) (26). Each data set was normalized by upper quartile normalization, and then transcript abundance was quantified using reads assigned per the kilobase of target per million mapped reads (RPKM) normalization method. Differential gene expression was assessed in Rockhopper using local regression with an error term modelled with a negative binomial distribution. Since multiple testing was performed, *q* values were reported, which reflect *P* values adjusted using the Benjamini-Hochberg procedure (25). Selection criteria for differential expression required genes to have a fold change greater than 1.5 and a *q* value of less than 0.05.

Owing to the high biological variability, OPLS-DA was conducted using the *ropls* package (69) in R software (version 3.2.3) (70). Differentially expressed genes resulting from the previous analysis were filtered to include only genes with VIP scores of at least 1.5, a corresponding weight (the relative degree of influence) cutoff to be at least ≥0.02 or ≤−0.02, and a coefficient value to be at least ≥0.001 or ≤−0.001.

### RT-qPCR.

cDNA was synthesized from the RNA previously extracted from PA14 grown in the blood of three TPs and three HVs using the QuantiTect reverse transcription kit (Qiagen). A 200-ng aliquot of cDNA was mixed with SYBR green PCR master mix (Life Technologies) and 250 nM specific primer. Amplification and detection of the product were carried out using the StepOne Plus real-time PCR system (Life Technologies). Three independent biological replicates of RNA samples were used for each experiment. In addition, each PCR was set up in technical triplicate. The quantity of cDNA in different samples was normalized using 30S rRNA (*rpsL*) as an internal standard. Gene expression analysis was performed using StepOne Plus software, version 2.2.2 (Life Technologies).

### Murine model of thermal injury.

Adult female Swiss Webster mice (Charles River, Wilmington, MA) weighing ∼23 g were utilized in the murine model of thermal injury (33). Briefly, mice were anesthetized, their backs were shaved, and the mice were placed in a template that exposes approximately 15% of the total body surface area. Thermal injury was induced by placing the exposed surface in 90°C water for 8 s, which induces a nonlethal, third-degree burn (33). For comparison of PA14 and PA14::*mcdA* strains, mice were injected with 200 CFU of either strain and observed for mortality for 2 days postinfection. At 24 h after thermal injury and infection, mice were euthanized and the livers and spleens were extracted, homogenized, diluted, and plated for recovery of CFU/g of tissue as previously described (33).

For analysis of expression of *mdcA*, *PA14_02570*, *mdcC*, and *mdcD* in PA14 grown in the blood of thermally injured mice, three mice were injured and three were left uninjured. At 12 h after thermal injury, the mice were anesthetized and blood was collected via cardiac puncture. The blood was inoculated with PA14 as described in “Growth of P. aeruginosa strains in whole blood” above.

Animals were treated humanely and in accordance with the protocol approved by the Animal Care and Use Committee at TTUHSC, Lubbock, TX.

### GC-TOF MS and data analysis.

Serum samples were submitted to the West Coast Metabolomics Center at the University of California, Davis, CA, for metabolomic analysis. The untargeted metabolite profiling approach was undertaken using GC-TOF MS according to the method reported by Fiehn et al. (71). Of the 439 detected metabolites, 135 were identified using the MassBank of North America and methods previously described (71).

A table of detected metabolites and their corresponding peak intensities was received and analyzed by first normalizing peak intensities by vector normalization (72); relative peak intensities then were log transformed and scaled by pareto scaling using the MetaboAnalyst program (http://metaboanalyst.ca/; accessed 20 November 2018) (40). We used Student's *t* test to assess the statistical significance of metabolite abundance between the two groups. For the multivariate statistical analysis of the metabolite data by PCA and clustering analysis, the preprocessed and normalized data set was imported into the ClustVis web tool (https://biit.cs.ut.ee/clustvis/; accessed 20 November 2018) and further analyzed (73).

### Analysis of iron levels in the sera of TPs and HVs.

The level of iron in the sera from TPs and HVs was assessed indirectly through its binding to pyoverdine (61, 62). As an alternative to purified pyoverdine, we separated pyoverdine-enriched supernatant (PES) from PA14 grown in an iron-deficient medium. PA14 was grown overnight in LB broth and then subcultured twice over 48 h in the iron-depleted complex medium Trypticase soy broth dialysate supplemented with 1% glycerol and 50 mM monosodium glutamate (74). Cultures were incubated at 37°C with shaking at 250 rpm. After the second subculture, the bacterial cells were pelleted by centrifugation at 10,000 × *g* for 15 min and the clear PES samples were utilized for analysis.

A standard curve was prepared by adding 100 μl of serial dilutions of ferric chloride (FeCl_3_) to 1 ml PES, followed by incubation at room temperature for 10 min to allow the iron-siderophore complex to form. Absorbance was measured at 460 nm as previously described (61, 62). To analyze iron levels in the sera of the TPs and HVs, 100 μl of the serum to be tested was added to 1 ml of PES, mixtures were incubated at room temperature for 10 min, and the *A*_460_ was obtained. The equivalent amount of iron within the sera was calculated using the standard curve.

### Data availability.

The raw RNA-seq data sets generated during this study are available through NCBI’s BioProject database under accession number PRJNA436010. The previously published BioProject accession number PRJNA287707 was also used (17). The metabolomics data presented in this study are available through the Metabolomics Workbench public repository under accession number ST001131. (75).
