# Supplementary figures and images for: Pseudomonas aeruginosa Alters Its Transcriptome Related to Carbon Metabolism and Virulence as a Possible Survival Strategy in Blood from Trauma Patients
Source: mSystems. 2019 May 7;4(4):e00312-18. doi: 10.1128/mSystems.00312-18 (PMC6506614; doi:10.1128/mSystems.00312-18)

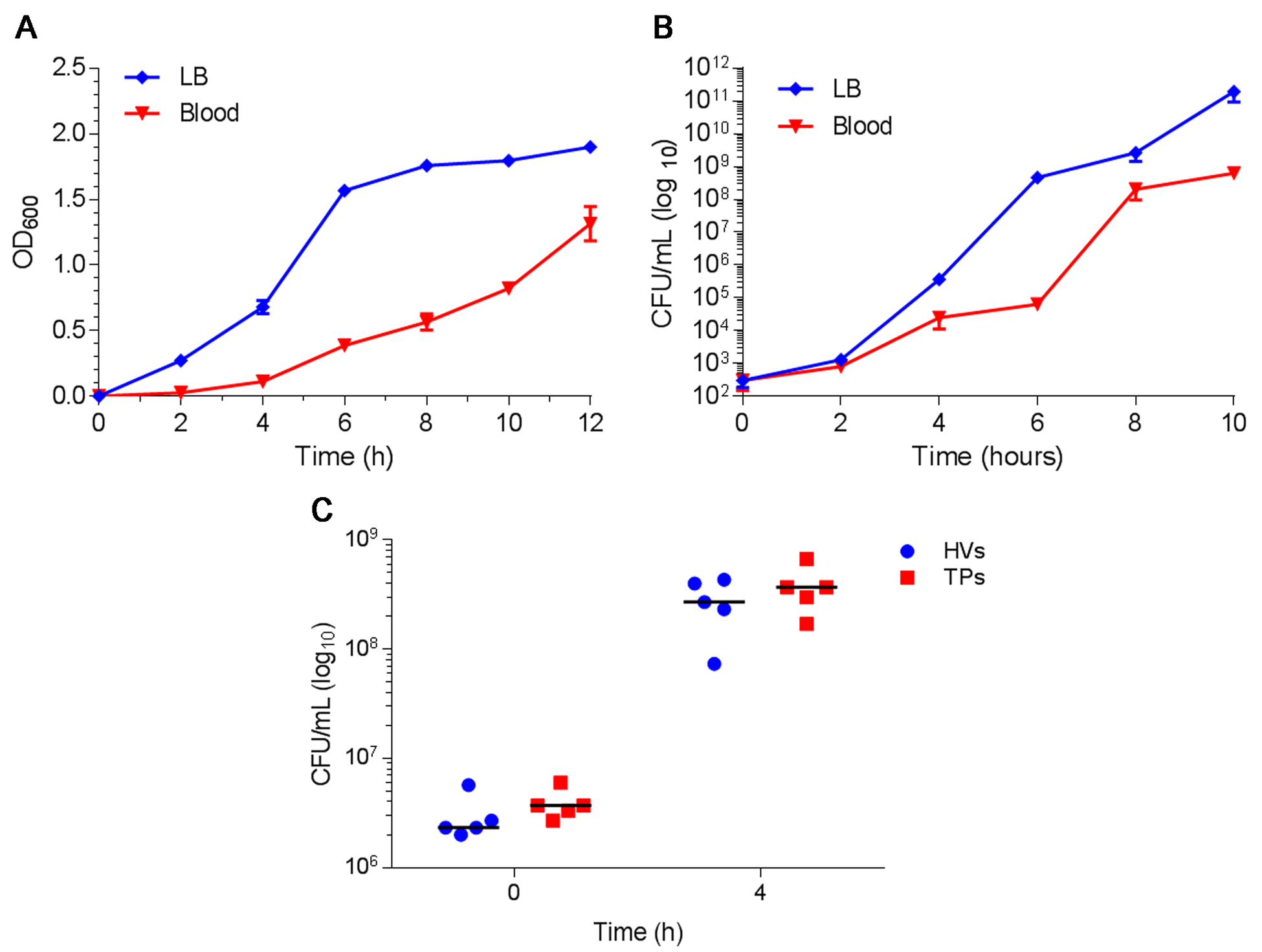

Supplement: FIG S1 [file mSystems.00312-18-sf001.tif]

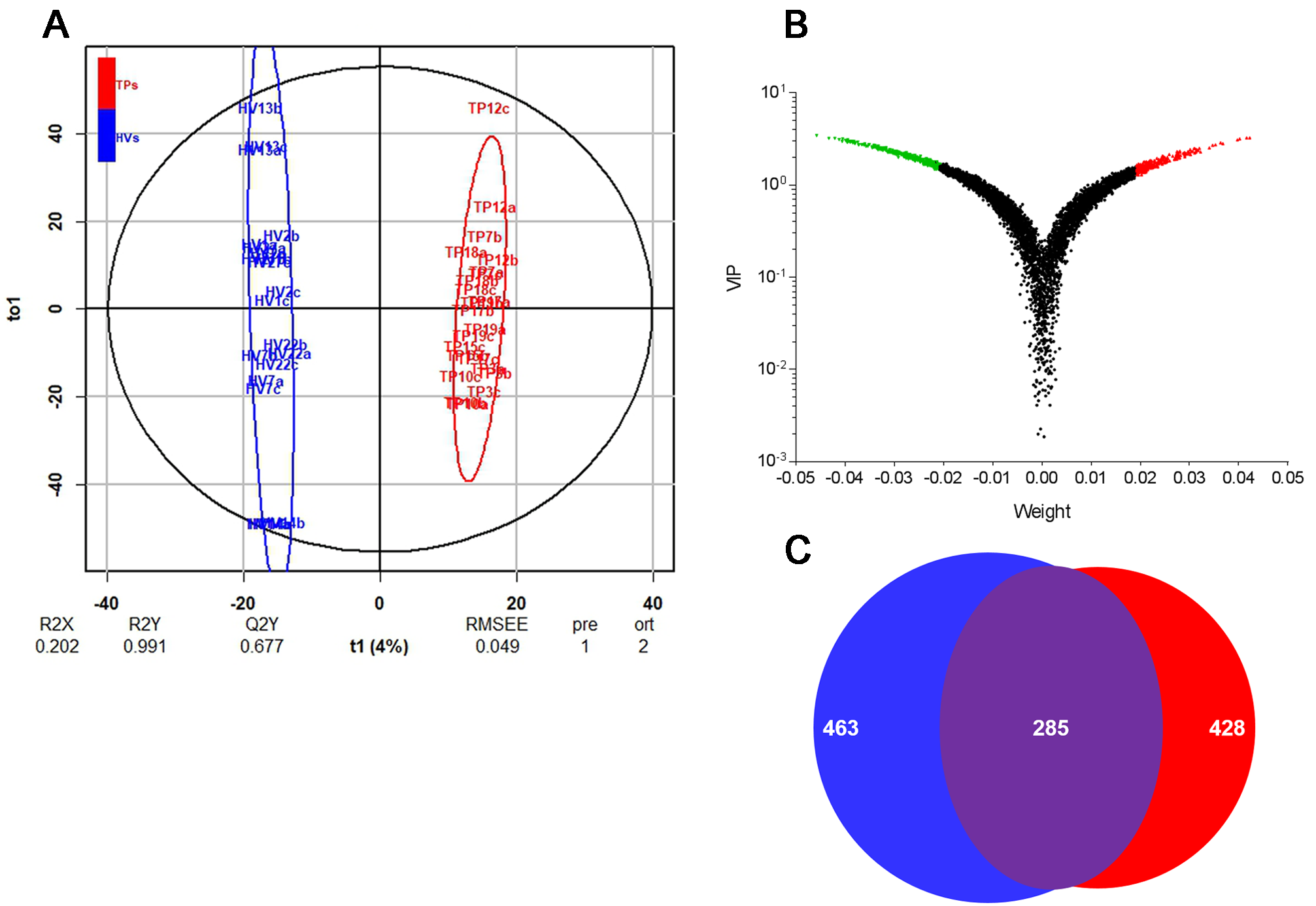

Supplement: FIG S2 [file mSystems.00312-18-sf002.tif]

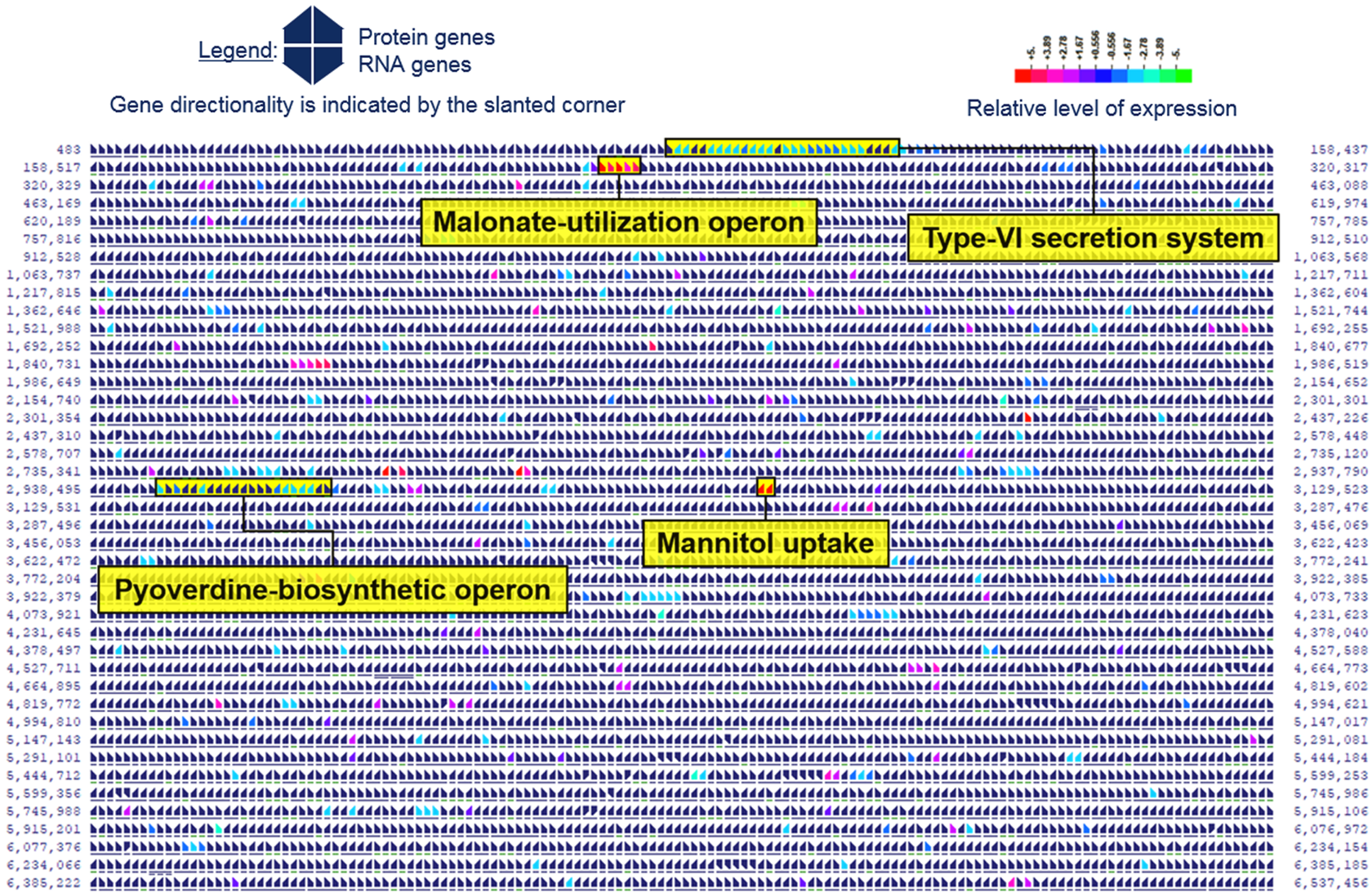

Supplement: FIG S3 [file mSystems.00312-18-sf003.tif]

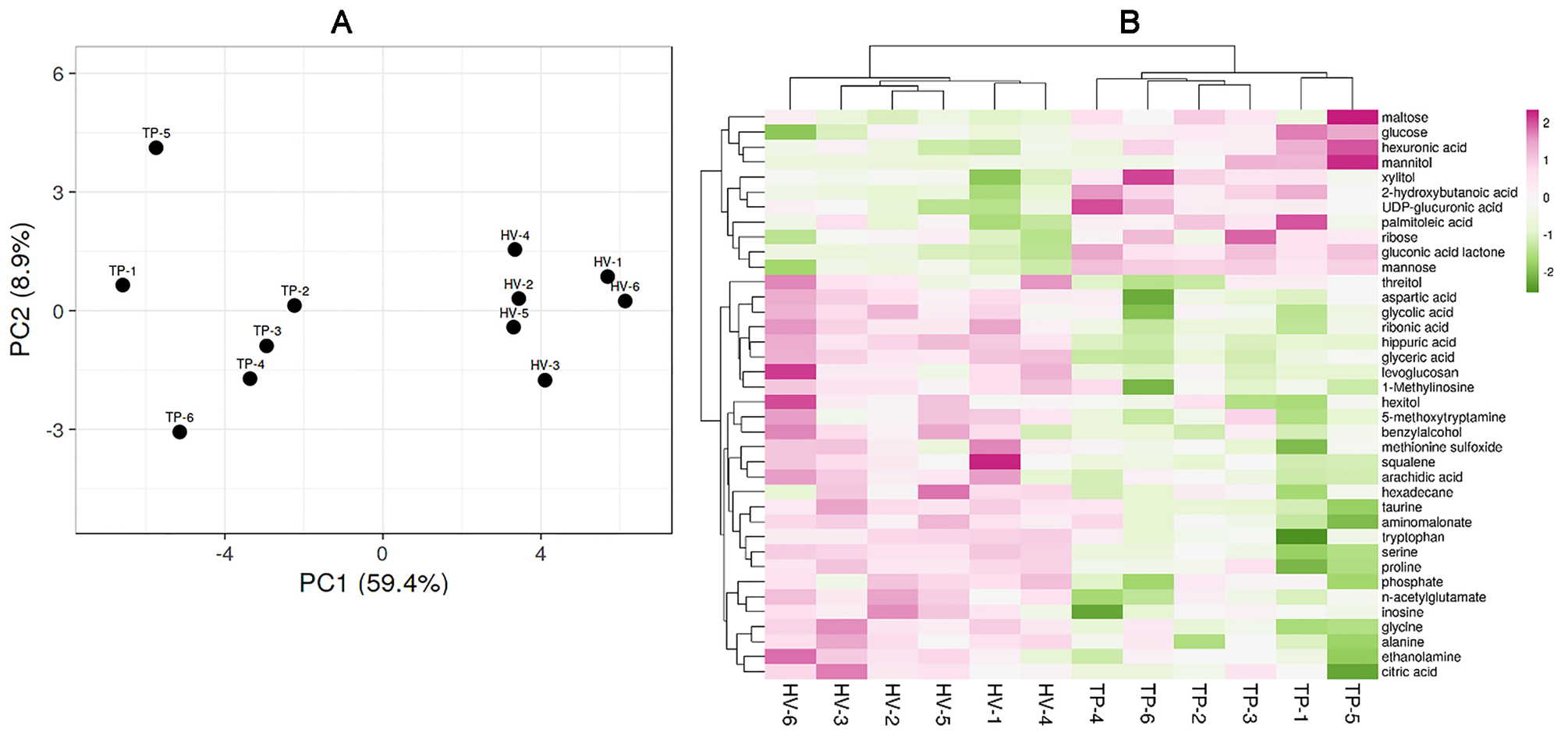

Supplement: FIG S4 [file mSystems.00312-18-sf004.tif]

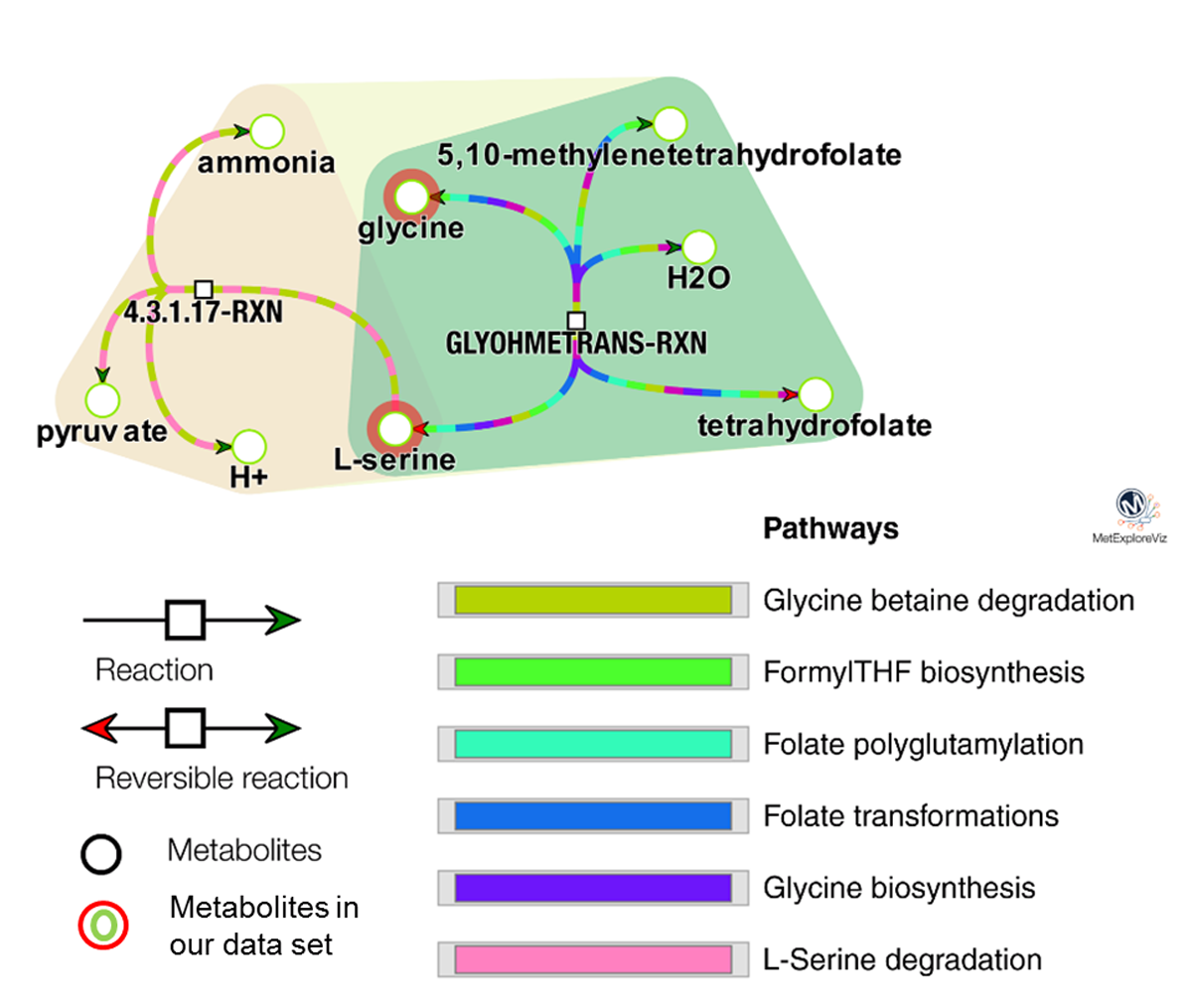

Supplement: FIG S5 [file mSystems.00312-18-sf005.tif]

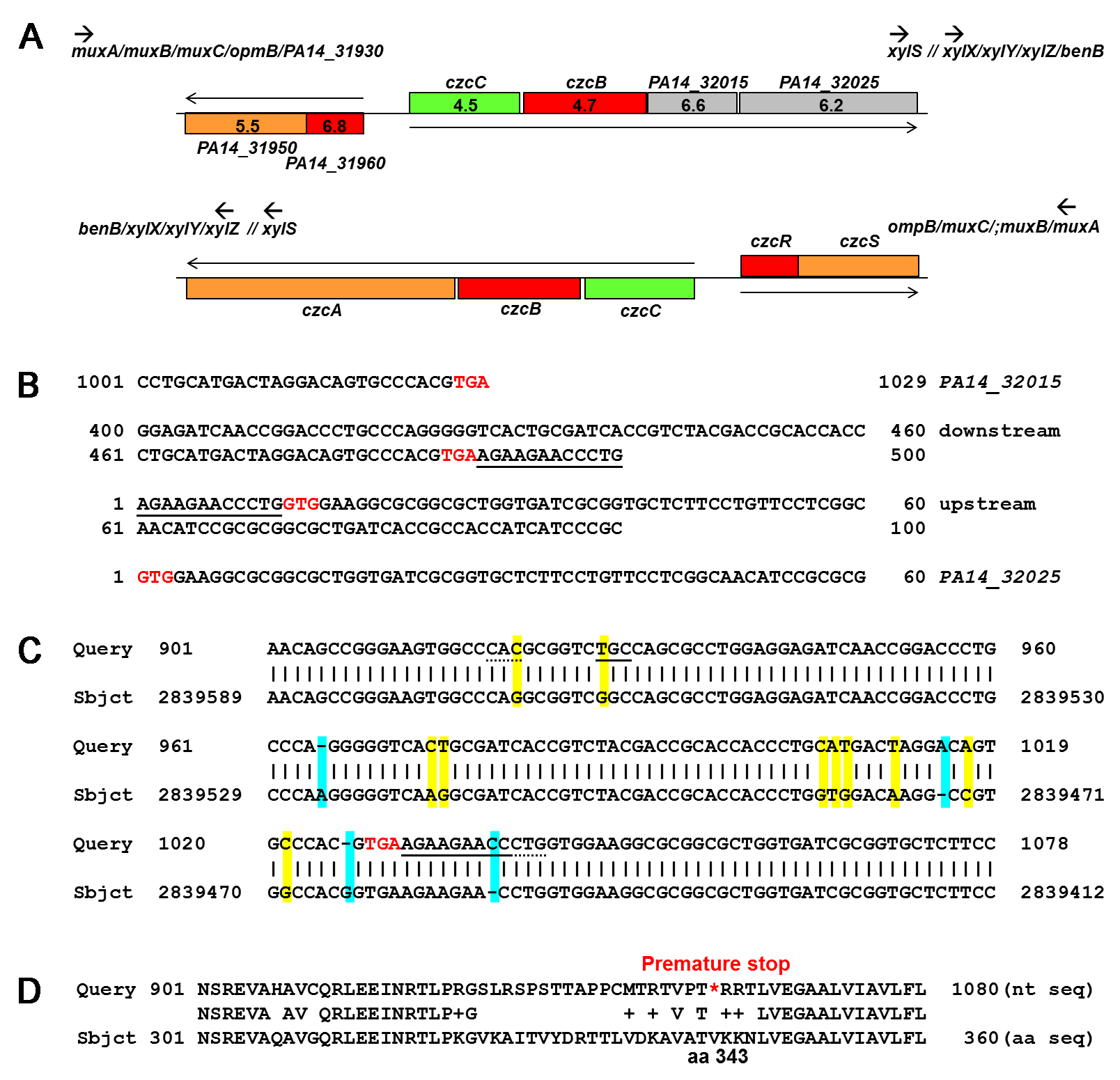

Supplement: FIG S6 [file mSystems.00312-18-sf006.tif]
