# Supplementary material for: Pseudomonas aeruginosa Alters Its Transcriptome Related to Carbon Metabolism and Virulence as a Possible Survival Strategy in Blood from Trauma Patients
Source: mSystems. 2019 May 7;4(4):e00312-18. doi: 10.1128/mSystems.00312-18 (PMC6506614; doi:10.1128/mSystems.00312-18)
Supplement: TABLE S1 [file mSystems.00312-18-st001.pdf]

Table S1

Selected genes exemplifying significant upregulation (red), downregulation (green), or no significant change.[a]

	Expression		Fold Change								
Locus	HVs	TPs	≥1.50 (up)	≥-1.50 (down)	*p* Value ≤ 0.05[b]	*q* Value ≤ 0.05[b]	Weight ≥ 0.02 ≤ −0.02	VIP ≥ 1.5	Coefficient ≥ 0.001 ≤ −0.001	Pass / Fail[c]	Failure criteria[d]
PA14_01500	8	10	1.3		0.03	1.00	0.01	0.3	0.000	fail	fc[e]
PA14_01510	31	22		-1.41	0.00	0.02	-0.01	1.2	-0.001	fail	fc
PA14_01520	31	31	1.00		0.55	1.00	0.00	0.2	0.000	fail	fc
PA14_01540	112	85		-1.32	0.00	0.03	-0.02	1.5	-0.001	fail	fc
PA14_01550	66	63	0.95		0.71	1.00	0.00	0.1	0.000	fail	fc
PA14_01560	94	138	1.47		0.00	0.00	0.02	1.7	0.001	fail	fc
PA14_01580	89	50		-1.78	0.00	0.00	-0.03	2.1	-0.002	pass	
PA14_01600	170	116		-1.47	0.00	0.00	-0.02	1.7	-0.001	fail	fc
PA14_01610	110	73		-1.51	0.00	0.00	-0.02	1.8	-0.002	pass	
PA14_01620	159	137		-1.16	0.16	0.57	-0.01	0.7	-0.001	fail	fc
PA14_01640	12	11		-1.09	0.99	1.00	0.00	0.1	0.000	fail	fc
PA14_01660	15	20	1.33		0.00	0.00	0.02	1.4	0.001	fail	fc
PA14_01670	0	0	0.00	0.00	0.54	1.00	0.00	0.3	0.000	fail	fc
PA14_01680	1	0	0.00	0.00	0.16	1.00	-0.01	0.5	0.000	fail	fc
PA14_01690	0	0	0.00	0.00	0.00	1.00	-0.01	0.8	-0.001	fail	fc
PA14_02500	1	3	3.00		0.00	1.00	0.01	1.3	0.001	fail	q, w, v
PA14_02510	2	3	1.50		0.07	1.00	0.00	0.3	0.000	fail	all
PA14_02520	626	268		-2.34	0.00	0.00	-0.03	2.1	-0.002	pass	
PA14_02530	116	174	1.50		0.00	0.00	0.02	1.8	0.002	pass	
PA14_02550	44	258	5.86		0.00	0.00	0.03	2.2	0.002	pass	
PA14_02560	5	21	4.20		0.00	0.00	0.03	2.0	0.002	pass	
PA14_02570	4	24	6.00		0.00	0.00	0.02	1.8	0.002	pass	
PA14_02580	12	36	3.00		0.00	0.00	0.03	1.9	0.002	pass	
PA14_02590	6	24	4.00		0.00	0.00	0.03	2.2	0.002	pass	
PA14_03100	2	5	2.50		0.00	1.00	0.02	1.3	0.001	fail	q, v
PA14_03110	14	8		-1.75	0.00	1.00	-0.01	1.1	-0.001	fail	q, w, v
PA14_03120	17	17	1.00		0.38	1.00	0.00	0.1	0.000	fail	fc
PA14_03130	8	13	1.63		0.00	1.00	0.02	1.5	0.001	fail	q
PA14_03150	22	25	1.14		0.04	0.19	0.01	0.3	0.000	fail	fc

PA14_03160	36	38	1.06		0.49	1.00	0.00	0.0	0.000	fail	fc
PA14_03163	477	569	1.19		0.00	0.01	0.02	1.2	0.001	fail	fc
PA14_03166	101	149	1.48		0.00	0.00	0.02	1.7	0.002	pass	
PA14_03170	19	22	1.16		0.01	0.06	0.01	0.6	0.001	fail	fc
PA14_03180	157	143		1.10	0.53	1.00	-0.01	0.4	0.000	fail	fc
PA14_03190	93	60		-1.55	0.00	0.00	-0.03	2.3	-0.002	pass	
PA14_03200	57	39		-1.46	0.00	0.00	-0.02	1.8	-0.002	pass	
PA14_03210	38	23		-1.65	0.00	0.00	-0.02	1.8	-0.002	pass	
PA14_03220	43	26		-1.65	0.00	0.00	-0.03	2.6	-0.002	pass	
PA14_03240	202	189		-1.07	0.88	1.00	0.00	0.4	0.000	fail	fc
PA14_03250	64	64	1.00		0.50	1.00	0.00	0.1	0.000	fail	fc
PA14_03265	176	144		-1.22	0.01	0.08	-0.01	1.1	-0.001	fail	fc
PA14_03270	120	108		-1.11	0.41	1.00	-0.01	0.8	-0.001	fail	fc
PA14_03285	50	54	1.08		0.09	0.37	0.01	0.4	0.000	fail	fc
PA14_03290	100	119	1.19		0.01	0.06	0.01	0.6	0.001	fail	fc
PA14_03300	32	39	1.22		0.01	0.04	0.01	0.9	0.001	fail	fc
PA14_32810	4	2		-2.00	0.01	1.00	-0.01	1.0	-0.001	fail	q, w, v
PA14_32820	8	11	1.38		0.00	1.00	0.01	0.7	0.001	fail	fc
PA14_32830	40	24		-1.67	0.00	0.00	-0.02	1.5	-0.001	pass	
PA14_32840	50	38		-1.32	0.00	0.02	-0.01	0.7	-0.001	fail	fc
PA14_32850	48	38		-1.26	0.01	0.08	-0.01	0.7	-0.001	fail	fc
PA14_32860	20	18		-1.11	0.69	1.00	0.00	0.3	0.000	fail	fc
PA14_32880	16	11		-1.45	0.00	1.00	-0.02	1.6	-0.001	fail	q
PA14_32890	129	129	1.00		0.86	1.00	0.00	0.2	0.000	fail	fc
PA14_32905	137	59		-2.32	0.00	0.00	-0.03	2.2	-0.002	pass	
PA14_32930	31	20		-1.55	0.00	0.00	-0.02	1.7	-0.001	pass	
PA14_32940	15	16	1.07		0.39	1.00	0.00	0.2	0.000	fail	fc
PA14_32950	3	2		-1.50	0.37	1.00	-0.01	0.4	0.000	fail	q, v
PA14_32970	5	3		-1.67	0.00	1.00	-0.01	1.1	-0.001	fail	q, w, v
PA14_32985	266	176		-1.51	0.00	0.00	-0.02	1.8	-0.002	pass	
PA14_33000	220	113		-1.95	0.00	0.00	-0.04	2.9	-0.002	pass	
PA14_33010	109	63		-1.73	0.00	0.00	-0.03	2.3	-0.002	pass	
PA14_33030	36	18		-2.00	0.00	0.00	-0.04	2.7	-0.002	pass	
PA14_33040	51	33		-1.55	0.00	0.00	-0.02	1.6	-0.001	pass	
PA14_33050	40	50	1.25		0.00	0.02	0.01	0.8	0.001	fail	fc
PA14_33060	9	11	1.22		0.01	1.00	0.01	0.5	0.000	fail	fc

PA14_33070	8	7		-1.14	0.59	1.00	0.00	0.4	0.000	fail	fc
PA14_33080	11	13	1.18		0.05	1.00	0.01	0.3	0.000	fail	fc
PA14_36100	9	4		-2.25	0.00	1.00	-0.03	2.0	-0.002	fail	q
PA14_36110	3	1		-3.00	0.00	1.00	-0.02	1.9	-0.002	fail	q
PA14_36120	4	3		-1.33	0.09	1.00	-0.01	0.3	0.000	fail	fc
PA14_36130	2	2	1.00		0.70	1.00	0.00	0.3	0.000	fail	fc
PA14_36150	0	0	0.00	0.00	0.00	1.00	-0.01	0.7	-0.001	fail	fc
PA14_36170	0	0	0.00	0.00	0.34	1.00	0.00	0.2	0.000	fail	fc
PA14_36180	13	15	1.15		0.03	1.00	0.01	0.5	0.000	fail	fc
PA14_36190	17	10		-1.70	0.00	1.00	-0.02	1.4	-0.001	fail	q, v
PA14_36200	315	299		-1.05	0.74	1.00	0.00	0.0	0.000	fail	fc
PA14_36220	6	9	1.50		0.00	1.00	0.01	0.9	0.001	fail	q, w, v
PA14_36230	6	10	1.67		0.00	1.00	0.01	0.9	0.001	fail	q, w, v
PA14_36250	7	11	1.57		0.00	1.00	0.01	0.9	0.001	fail	q, w, v
PA14_36260	2	2	1.00		0.02	1.00	0.01	0.4	0.000	fail	fc
PA14_36270	25	27	1.08		0.16	0.55	0.00	0.4	0.000	fail	fc
PA14_36280	15	15	1.00		0.43	1.00	0.00	0.2	0.000	fail	fc
PA14_36290	17	31	1.82		0.00	0.00	0.03	2.4	0.002	pass	
PA14_36300	22	37	1.68		0.00	0.00	0.03	2.0	0.002	pass	

[a]Values were rounded to significant decimal before applying criteria.

[b]HVs versus TPs

[c]Genes were considered significantly altered in expression if all the criteria were met for q value, VIP (variable importance in projection) score, weight, and coefficient.

[d]fc, fold-change; q, q value; w, weight; v, VIP

[e]fc takes precedence; other criteria not withstanding
