# Supplementary material for: Pseudomonas aeruginosa Alters Its Transcriptome Related to Carbon Metabolism and Virulence as a Possible Survival Strategy in Blood from Trauma Patients
Source: mSystems. 2019 May 7;4(4):e00312-18. doi: 10.1128/mSystems.00312-18 (PMC6506614; doi:10.1128/mSystems.00312-18)
Supplement: TABLE S2 [file mSystems.00312-18-st002.pdf]

Table S2

The expression levels of the differentially expressed genes (TPs vs. HVs) and their product description and subclassification.

Locus	Gene	FC[a]	Product /Function[b] [function][c]	Protein Class Assignment[d]	Subclass[d]
PA14_00820[e]	*tagQ1*	-1.70	TagQ1 / [Type VI secretion-associated lipoprotein TagQ]	Membrane proteins; Protein secretion/ export apparatus	T6SS, HS1-T6SS
PA14_00830	*tagR1*	-1.70	TagR1 / [Type VI secretion posttranslational regulatory protein TagR]	Hypothetical, unclassified, unknown	T6SS, HS1-T6SS
PA14_00875	*ppkA*	-1.84	PpkA / Serine/threonine protein kinase	Adapation, protection; Protein secretion/ export apparatus; Translation, post-translational modification, degradation	T6SS, HS1-T6SS
PA14_00890	*pppA*	-1.93	PppA / [serine/threonine phosphoprotein phosphatase]	Protein secretion/export apparatus; Putative enzymes	T6SS, HS1-T6SS
PA14_00900	*tagF1*	-1.74	TagF / [Type VI secretion-associated protein]	Protein secretion/export apparatus	T6SS, HS1-T6SS
PA14_00910	*icmF1*	-1.71	IcmF1 / [ImcF domain-containing protein, type VI secretion]	Hypothetical, unclassified, unknown	T6SS, HS1-T6SS
PA14_00925	*tssL1*	-1.54	TssL1 / [Type IV / VI secretion system, DotU]	Hypothetical, unclassified, unknown	T6SS, HS1-T6SS
PA14_00940	*tssK1*	-1.70	TssK1 / [Type VI secretion protein]	Hypothetical, unclassified, unknown	T6SS, HS1-T6SS
PA14_00960	*tssJ1*	-1.82	TssJ1 / [Type VI secretion lipoprotein]	Fatty acid and phospholipid metabolism	T6SS, HS1-T6SS
PA14_00970	*PA14_00970*	-1.67	/ [Type VI secretion system, FHA domain-containing protein]	Hypothetical, unclassified, unknown	T6SS, HS1-T6SS
PA14_00990	*tssA1*	-1.85	TssA1 / [Type VI secretion-associated protein, ImpA family]	Hypothetical, unclassified, unknown	T6SS, HS1-T6SS
PA14_01010	*hsiB1*	-1.79	HsiB1 / [Type VI secretion protein TssB1 or ImpB]	Protein secretion/export apparatus	T6SS, HS1-T6SS
PA14_01020	*hsiC1*	-1.75	HsiC1 / [Type VI secretion protein TssB1, EvpB or ImpC]	Protein secretion/export apparatus	T6SS, HS1-T6SS
PA14_01030	*hcp1*	-1.57	Hcp1 / [Hcp1 family type VI secretion system effector]	Hypothetical, unclassified, unknown	T6SS, HS1-T6SS
PA14_01040	*tagJ1*	-1.52	TagJ1 / [Type VI secretion virulence protein, SciE type]	Protein secretion/export apparatus	T6SS, HS1-T6SS
PA14_01060	*tssE1*	-1.64	TssE1 / [Type VI secretion system lysozyme-like protein TssE]	Protein secretion/export apparatus	T6SS, HS1-T6SS
PA14_01070	*tssF1*	-1.56	TssF1 / [Type VI secretion protine TssF or ImpG]	Protein secretion/export apparatus	T6SS, HS1-T6SS

Locus	Gene	FC[a]	Product /Function[b] [function][c]	Protein Class Assignment[d]	Subclass[d]
PA14_01080	*tssG1*	-1.82	TssG1 / [Type VI secretion protein TssG]	Hypothetical, unclassified, unknown	T6SS, HS1-T6SS
PA14_01100	*clpV1*	-1.77	ClpV1 / [Type VI secretion ATPase ClpV1]	Translation, post-translational modification, degradation	T6SS, HS1-T6SS
PA14_01110	*vgrG1*	-1.77	VgrG1 / [Type VI secretion system, Vgr family or ImpA family]	Hypothetical, unclassified, unknown	T6SS, HS1-T6SS
PA14_01150	*eagT6*	-2.00	EagT6 / Hypothetical protein	Hypothetical, unclassified, unknown	T6SS, HS1-T6SS
PA14_01160	*PA14_01160*	-1.77	/ [ImpA family type VI secretion-associated protein; Rhs element Vgr protein]	Hypothetical, unclassified, unknown	T6SS
PA14_01180	*PA14_01180*	-1.63	/ Hypothetical protein	Hypothetical, unclassified, unknown	
PA14_01200	*PA14_01200*	-1.64	/ Hypothetical protein	Hypothetical, unclassified, unknown	
PA14_01460	*dctA*	-1.54	DctA / C4-dicarboxylate transport protein	Transport of small molecules	
PA14_01580	*gabP*	-1.78	GabP / Gamma-aminobutyrate (GABA) permease	Transport of small molecules	
PA14_01610	*PA14_01610*	-1.51	/ Hypothetical protein [cupin 2 protein]	Carbon compound catabolism	
PA14_02230	*cheW*	-1.83	CheW / Purine-binding chemotaxis protein	Chemotaxis	
PA14_02260	*PA14_02260*	-1.73	/ Two-component response regulator [CheY]	Two-component regulatory systems	
PA14_02520	*PA14_0*	-2.34	/ Hypothetical protein	Hypothetical, unclassified, unknown	
PA14_02530	*PA14_02530*	1.50	/ Hypothetical protein [esterase or alpha/beta hydrolase fold protein]	Hypothetical, unclassified, unknown	
PA14_02550	*mdcA*	5.86	MdcA / Malonate decarboxylase subunit alpha	Carbon compound catabolism	Malonate
PA14_02560	*PA14_02560*	4.20	/ Triphosphoribosyl-dephospho-CoA synthase	Biosynthesis of cofactors, prosthetic groups and carriers	Malonate
PA14_02570	*mdcC*	6.00	MdcC / Malonate decarboxylase subunit delta	Carbon compound catabolism	Malonate
PA14_02580	*mdcD*	3.00	MdcD / Malonate decarboxylase subunit beta	Carbon compound catabolism	Malonate
PA14_02590	*mdcE*	4.00	MdcE / Malonate decarboxylase subunit gamma	Carbon compound catabolism	Malonate
PA14_03190	*tli3*	-1.55	Tli3 / Hypothetical protein; probably T6SS related	Hypothetical, unclassified, unknown	T6SS
PA14_03210	*PA14_03210*	-1.65	/ Hypothetical protein; probably T6SS related	Hypothetical, unclassified, unknown	T6SS

Locus	Gene	FC[a]	Product /Function[b] [function][c]	Protein Class Assignment[d]	Subclass[d]
PA14_03220	*vgrG2b*	-1.65	VgrG2b / [ImpA family type VI secretion-associated protein; Rhs element Vgr protein]	Hypothetical, unclassified, unknown	T6SS
PA14_03610	*PA14_03610*	-1.71	/ Zn-dependent protease with chaperone function	Chaperones and heat shock proteins	
PA14_03700	*sbp*	2.02	Sbp / Sulfate-binding protein	Transport of small molecules	
PA14_03710	*PA14_03710*	2.04	/ Hypothetical protein	Hypothetical, unclassified, unknown	
PA14_03790	*PA14_03790*	-1.54	/ Sensory box GGDEF domain-containing protein	Two-component regulatory system	
PA14_04180	*PA14_04180*	3.04	/ Hypothetical protein [stress-induced protein YgiW]	Hypothetical, unclassified, unknown	Heavy metals
PA14_04290	*PA14_04290*	-1.87	/ [Arginine-specific autotransporter of *Pseudomonas aeruginosa* , AaaA]	Amino acid biosynthesis and metabolism; Membrane proteins	Amino acids
PA14_05110	*PA14_0* [illegible]	-1.54	/ Hypothetical protein [GTPase (dynamin- [illegible]	Hypothetical, unclassified, unknown	
PA14_05600	*PA14_05600*	-1.67	/ Hypothetical protein	Hypothetical, unclassified, unknown	
PA14_05620	*sahH*	-1.73	SahH / S-adenosyl-L-homocysteine hydrolase	Amino acid biosynthesis and metabolism; Central intermediary metabolism	Amino acids
PA14_06420	*PA14_06420*	-3.09	/ [LamB/YcsF family protein]	Antibiotic resistance and susceptibility	
PA14_06430	*PA14_06430*	-3.15	/ [acetyl-CoA carboxylase, bitoin carboxyl carrier protein AccB]	Putative enzymes	
PA14_06920	*PA14_06920*	-1.64	/ Class III pyridoxal phosphate-dependent aminotransferase	Putative enzymes; Transport of small molecules	
PA14_07090	*metK*	-1.80	MetK / S-adenosylmethionine synthetase	Amino acid biosynthesis and metabolism; Antibiotic resistance and susceptibility; Central intermediary metabolism	Amino acids
PA14_07330	*PA14_07330*	-1.55	/ Hypothetical protein	Hypothetical, unclassified, unknown	
PA14_07355	*PA14_07355*	1.93	/ [Alkylhydroperoxidase or carboxymucono-lactone decarboxylase family protein]	Hypothetical, unclassified, unknown	
PA14_07400	*PA14_07400*	-1.56	/ Hypothetical protein	Hypothetical, unclassified, unknown	
PA14_08450	*PA14_08450*	-1.53	/ Hypothetical protein	Membrane proteins	
PA14_09280	*pchF*	-1.56	PchF / Pyochelin synthetase	Transport of small molecules	Pyochelin

Locus	Gene	FC[a]	Product /Function[b] [function][c]	Protein Class Assignment[d]	Subclass[d]
PA14_09300	*PA14_09300*	-1.80	/ ABC transporter ATP-binding protein	Transport of small molecules	Pyochelin
PA14_09320	*PA14_09320*	-1.81	/ ABC transporter ATP-binding protein	Transport of small molecules	Pyochelin
PA14_11370	*PA14_11370*	-1.82	/ Lipoprotein	Fatty acid and phospholipid metabolism	
PA14_11410	*ribC*	-1.75	RibC / Riboflavin synthase subunit alpha	Biosynthesis of cofactors, prosthetic groups and carriers	
PA14_11470	*pgpA*	1.62	PgpA / Phosphatidylglycerophosphatase A	Fatty acid and phospholipid metabolism	
PA14_12570	*PA14_12570*	-1.69	/ Transcriptional regulator, AsnC family	Transcriptional regulators	
PA14_13010	*PA14_13010*	3.08	/ [NLPA lipoprotein or methionine ABC transporter substrate-binding protein]	Hypothetical, unclassified, unknown	
PA14_13130	*PA14_13130*	-1.59	/ Hypothetical protein [type V secretory pathway, adhesin AidA]	Hypothetical, unclassified, unknown	
PA14_13140	*PA14_1*	-1.93	/ Hypothetical protein	Hypothetical, unclassified, unknown	
PA14_13230	*moaC*	-1.52	MoaC / Molybdenum cofactor biosynthesis protein	Biosynthesis of cofactors, prosthetic groups and carriers	
PA14_13300	*PA14_13300*	1.74	/ Collagenase-like protease	Translation, post-translational modification, degradation	
PA14_13580	*PA14_13580*	1.90	/ ABC transporter ATP-binding protein [Glycine betaine/L-proline transport ATP-binding protein]	Transport of small molecules	
PA14_14210	*PA14_14210*	-1.73	/ Hypothetical protein	Hypothetical, unclassified, unknown	
PA14_14290	*PA14_14290*	-2.19	/ [Glutathione S-transferase family protein]	Hypothetical, unclassified, unknown	
PA14_14390	*PA14_14390*	-1.50	/ ABC-type transport protein, periplasmic	Transport of small molecules	
PA14_15030	*leuA*	-2.12	LeuA / 2-isopropylmalate synthase	Amino acid biosynthesis and metabolism	Amino acids
PA14_15350	*PA14_15350*	1.88	/ Integrase	Related to phage, transposon, or plasmid	
PA14_16010	*PA14_16010*	2.29	/ [Long-chain acyl-CoA thioester hydrolase]	Fatty acid and phospholipid metabolism	
PA14_16180	*PA14_16180*	-1.68	/ Hypothetical protein	Hypothetical, unclassified, unknown	

Locus	Gene	FC[a]	Product /Function[b] [function][c]	Protein Class Assignment[d]	Subclass[d]
PA14_16190	*PA14_16190*	-1.57	/ Hypothetical protein	Hypothetical, unclassified, unknown	
PA14_16200	*PA14_16200*	-1.66	/ [Phosphatidylserine/phosphatidylglycerophosphate/cardiolipin synthases,related enzymes]	Hypothetical, unclassified, unknown	
PA14_16660	*PA14_16660*	2.88	/ Metal-transporting P-type ATPase	Transport of small molecules	Heavy metals
PA14_16840	*PA14_16840*	-1.74	/ Lipoprotein	Fatty acid and phospholipid metabolism	
PA14_17000	*PA14_17000*	-3.18	/ Hypothetical protein	Hypothetical, unclassified, unknown	
PA14_17120	*cdsA*	2.13	CdsA / Phosphatidate cytidylyltransferase	Fatty acid and phospholipid metabolism	
PA14_17220	*lpxB*	1.55	LpxB / Lipid-A-disaccharide synthase	Cell wall / LPS / capsule	
PA14_17340	*ispD*	1.52	IspD / 2-C-methyl-D-erythritol 4-phosphate cytidylyltransferase	Biosynthesis of cofactors, prosthetic groups and carriers	
PA14_17510	*PA14_17510*	-1.95	/ [putative TolB-like translocation protein]	Protein secretion/export apparatus	
PA14_17600	*PA14_17600*	-1.54	/ Hypothetical protein	Protein secretion/export apparatus	
PA14_17720	*PA14_17720*	-1.67	/ LuxR family transcriptional regulator	Transcriptional regulators	
PA14_17930	*glpD*	-1.60	GlpD / Glycerol-3-phosphate dehydrogenase	Central intermediary metabolism; Energy metabolism	
PA14_17980	*glpF*	-1.71	GlpF / Glycerol uptake facilitator protein	Transport of small molecules	
PA14_19020	*tse3*	-1.52	Tse3 / Hypothetical protein T6SS secreted effector protein	Secreted factors (toxins, enzymes, alginate)	T6SS
PA14_19100	*rhlA*	2.31	RhlA / Rhamnosyltransferase chain A	Secreted factors (toxins, enzymes, alginate)	
PA14_19190	*PA14_19190*	-1.59	/ Malate dehydrogenase	Central intermediary metabolism	
PA14_19320	*PA14_19320*	-2.17	/ Hypothetical protein	Hypothetical, unclassified, unknown	
PA14_19490	*lsfA*	2.37	LsfA / 1-Cys peroxiredoxin LsfA	Adaptation, protection	
PA14_19530	*PA14_19530*	3.11	/ NAD(P)H-dependent FMN reductase	Central intermediary metabolism	

Locus	Gene	FC[a]	Product /Function[b] [function][c]	Protein Class Assignment[d]	Subclass[d]
PA14_19680	*PA14_19680*	2.67	/ [Murein hydrolase transporter LgrA]	Membrane proteins; Protein secretion/ export apparatus	
PA14_20010	*hasR*	-2.27	HasR / Heme uptake outer membrane receptor	Transport of small molecules	Heme
PA14_20390	*phnJ*	4.75	PhnJ / [Carbon-phosphorus lyase complex subunit]	Transport of small molecules	
PA14_20550	*PA14_20550*	-1.71	/ Hypothetical protein	Hypothetical, unclassified, unknown	
PA14_21570	*PA14_21570*	2.99	/ Hypothetical protein	Hypothetical, unclassified, unknown	
PA14_21580	*PA14_21580*	2.78	/ Hypothetical protein	Hypothetical, unclassified, unknown	
PA14_21590	*PA14_2*	3.00	/ Hypothetical protein	Hypothetical, unclassified, unknown	
PA14_21600	*PA14_21600*	4.18	/ [DoxX family protein]	Membrane proteins	
PA14_21610	*oprO*	4.92	OprO / Pyrophosphate-specific outer membrane porin	Transport of small molecules	
PA14_22280	*PA14_2*	1.72	/ Pirin-related protein	Transcriptional regulators	
PA14_24050	*xcpV*	-1.68	XcpV / General secretion pathway protein I for T2SS	Protein secretion/export apparatus	T2SS
PA14_24300	*PA14_24300*	-1.56	/ Hypothetical protein	Hypothetical, unclassified, unknown	
PA14_24330	*PA14_24330*	-1.52	/ [RND efflux transporter]	Motility and attachment	
PA14_24850	*PA14_24850*	1.70	/ [Glutaredoxin or thiol-disulfide isomerase and thioredoxin]	Hypothetical, unclassified, unknown	
PA14_24950	*PA14_2*	-1.73	/ FAD-dependent glycerol-3-phosphate	Carbon compound catabolism; Energy	
PA14_24960	*PA14_24960*	-1.71	/ Carbohydrate kinase	Carbon compound catabolism	
PA14_25030	*PA14_2[illegible]*	1.65	/ Hypothetical protein	Hypothetical, unclassified, unknown	
PA14_25410	*PA14_2[illegible]*	-1.55	/ Hypothetical protein	Hypothetical, unclassified, unknown	
PA14_25540	*ptpA*	1.54	PtpA / Phosphotyrosine protein phosphatase	Amino acid biosynthesis and metabolism	Amino acids
PA14_25620	*PA14_25620*	1.72	/ Hypothetical protein	Hypothetical, unclassified, unknown	
PA14_25640	*plsX*	1.53	PlsX / Glycerol-3-phosphate acyltransferase	Fatty acid and phospholipid metabolism	

Locus	Gene	FC[a]	Product /Function[b] [function][c]	Protein Class Assignment[d]	Subclass[d]
PA14_25650	*fabD*	-1.52	FabD / Malonyl CoA-acyl carrier protein transacylase	Fatty acid and phospholipid metabolism	
PA14_25980	*aroF*	-2.90	AroF / Phospho-2-dehydro-3-deoxyheptonate aldolase	Amino acid biosynthesis and metabolism	Amino acids
PA14_26020	*PA14_26020*	-1.63	/ Aminopeptidase	Secreted factors (toxins, enzymes, alginate)	
PA14_27100	*lipA*	-1.78	LipA / Lactonizing lipase precursor	Secreted factors (toxins, enzymes, alginate)	
PA14_27520	*PA14_27520*	-1.56	/ Glutathione peroxidase	Adaptation, protection	
PA14_27830	*PA14_27830*	5.81	/ [Two-component response repressor, PtrA]	Adaptation, protection; Transcriptional regulators	
PA14_28010	*PA14_28010*	-1.68	/ Hypothetical protein	Hypothetical, unclassified, unknown	
PA14_28390	*PA14_28390*	-2.00	/ Hypothetical protein	Hypothetical, unclassified, unknown	
PA14_29190	*PA14_29190*	-1.70	/ Tsi2-like immunity protein	Adaptation, protection	T6SS
PA14_29200	*PA14_29200*	-1.79	/ Tse2-like effector protein	Secreted factors (toxins, enzymes, alginate)	T6SS
PA14_29330	*PA14_29330*	-1.91	/ Hypothetical protein	Hypothetical, unclassified, unknown	
PA14_29400	*PA14_29400*	-1.86	/ Hypothetical protein	Cell wall / LPS / capsule	
PA14_29830	*PA14_2*	-1.80	/ Methyltransferase	Antibiotic resistance and susceptibility	
PA14_30700	*PA14_30700*	1.53	/ Sensor/response regulator hybrid [Hpt sensor hybrid histidine kinase]	Two-component regulatory systems	
PA14_30730	*PA14_30730*	-1.52	/ Hypothetical protein	Hypothetical, unclassified, unknown	
PA14_30800	*PA14_30800*	1.60	/ Hypothetical protein	Energy metabolism	
PA14_31040	*PA14_31040*	1.50	/ Cation efflux system protein	Protein secretion/export apparatus	
PA14_31050	*PA14_31050*	1.68	/ Hypothetical protein [Heat shock protein HtpX]	Hypothetical, unclassified, unknown	
PA14_31580	*PA14_31580*	1.83	/ Acyl-CoA dehydrogenase	Fatty acid and phospholipid metabolism; Putative enzymes	

Locus	Gene	FC[a]	Product /Function[b] [function][c]	Protein Class Assignment[d]	Subclass[d]
PA14_31700	*PA14_3*	-1.71	/ CDP-alcohol phosphatidyltransferase	Fatty acid and phospholipid metabolism;	
PA14_31720	*PA14_3*	-2.38	/ [Lysophospholipase]	Fatty acid and phospholipid metabolism	
PA14_31750	*PA14_3 1750*	-1.78	/ Acyltransferase	Fatty acid and phospholipid metabolism	
PA14_31760	*PA14_3 1760*	-1.74	/ Phosphatidate cytidylyltransferase	Fatty acid and phospholipid metabolism	
PA14_31770	*PA14_3 1770*	-1.82	/ Oxidoreductase	Energy metabolism	
PA14_31820	*PA14_3 1820*	-2.30	/ Aminotransferase	Amino acid biosynthesis and metabolism	Amino acids
PA14_31960	*PA14_3 1960*	6.80	/ Two-component response regulator [CzrR/CzcR]	Two-component regulatory systems	Heavy metals
PA14_31990	*czcB*	4.50	CzcB / Cobalt/zinc/cadmium efflux RND	Transport of small molecules	Heavy metals
PA14_32250	*PA14_3*	5.17	/ Hypothetical protein	Hypothetical, unclassified, unknown	
PA14_32270	*PA14_3*	2.96	/ Porin [outer membrane porin, OprD family]	Transport of small molecules	
PA14_32905	*PA14_3*	-2.32	/ Hypothetical protein [enterochelin esterase-	Hypothetical, unclassified, unknown	
PA14_32930	*PA14_3*	-1.55	/ Hypothetical protein	Hypothetical, unclassified, unknown	
PA14_32985	*gcvH2*	-1.51	GcvH2 / Glycine cleavage system H protein	Amino acid biosynthesis and metabolism	Amino acids
PA14_33000	*gcvP2*	-1.95	GcvP2 / Glycine dehydrogenase	Amino acid biosynthesis and metabolism	Amino acids
PA14_33010	*glyA2*	-1.73	GlyA2 / Serine hydroxymethyltransferase	Amino acid biosynthesis and metabolism	Amino acids
PA14_33030	*sdaA*	-2.00	SdaA / L-serine dehydratase	Amino acid biosynthesis and metabolism	Amino acids
PA14_33040	*gcvT2*	-1.55	GcvT2 / Glycine cleavage system protein T2	Amino acid biosynthesis and metabolism; Central intermediary metabolism	Amino acids
PA14_33500	*pvdH*	-2.35	PvdH / Diaminobutyrate-2-oxoglutarate aminotransferase	Adaptation, protection; Transport of small molecules	Pyoverdine
PA14_33520	*PA14_3 3520*	-1.55	/ Thioesterase	Adaptation, protection; Biosynthesis of cofactors, prosthetic groups and carriers; Putative enzymes	
PA14_33550	*PA14_3 3550*	-1.83	/ ABC transporter ATP-binding protein	Transport of small molecules	
PA14_33680	*fpvA*	-1.56	FpvA / Ferripyoverdine receptor	Transport of small molecules	Pyoverdine
PA14_33690	*pvdE*	-2.11	PvdE / Pyoverdine biosynthesis protein	Adapatation, protection; Membrane proteins; Transport of small molecules	Pyoverdine
PA14_33700	*pvdF*	-1.89	PvdF / Pyoverdine synthetase	Adaptation, protection; Secreted factors (toxins, enzymes, alginate)	Pyoverdine
PA14_33710	*pvdO*	-1.83	PvdO / [Chromophore maturation protein]	Adaptation, protection	Pyoverdine

Locus	Gene	FC[a]	Product /Function[b] [function][c]	Protein Class Assignment[d]	Subclass[d]
PA14_33720	*pvdN*	-1.72	PvdN / [Chromophore maturation protein; class V aminotransferase	Adapatation, protection; Transport of small molecules	Pyoverdine
PA14_33750	*PA14_3* [illegible]	-1.59	/ Outer membrane protein [pyoverdine efflux]	Transport of small molecules	Pyoverdine
PA14_33810	*pvdA*	-2.27	PvdA / L-ornithine 5-monooxygenase	Biosynthesis of cofactors, prosthetic groups and carriers	Pyoverdine
PA14_33820	*pvdQ*	-1.72	PvdQ / Penicillin acylase-related protein	Adaptation, protection; Antibiotic resistance and susceptibility	Pyoverdine
PA14_33860	*lldA*	1.85	LldA / L-lactate dehydrogenase	Energy metabolism	
PA14_33870	*PA14_33870*	1.80	/ Hypothetical protein	Hypothetical, unclassified, unknown	
PA14_34030	*hcp3*	-2.67	Hcp3 / [Hemolysin co-regulated T6SS effector]	Hypothetical, unclassified, unknown	T6SS, H3-T6SS
PA14_34050	*hsiC3*	-2.00	HsiC3 / [EvpB family T6SS protein]	Hypothetical, unclassified, unknown	T6SS, H3-T6SS
PA14_34370	*PA14_34370*	9.60	/ ABC maltose/mannitol transporter ATP-binding protein	Transport of small molecules	Mannitol
PA14_34390	*PA14_34390*	34.00	/ Binding-protein-dependent maltose/mannitol transport protein	Transport of small molecules	Mannitol
PA14_34600	*PA14_34600*	1.52	/ Glyceraldehyde-3-phosphate dehydrogenase	Carbon compound catabolism	
PA14_35780	*PA14_35780*	-1.59	/ Hypothetical protein	Hypothetical, unclassified, unknown	
PA14_35790	*PA14_35790*	-1.53	/ Homospermidine synthase	Putative enzymes; Secreted factors (toxins, enzymes, alginate)	
PA14_36290	*PA14_36290*	1.82	/ [Oxidoreductase]	Energy metabolism; Putative enzymes	
PA14_36300	*PA14_36300*	1.68	/ TetR family transcriptional regulator	Transcriptional regulators	
PA14_36330	*hcnA*	2.94	HcnA / Hydrogen cyanide synthase	Central intermediary metabolism	
PA14_37080	*PA14_37080*	-1.52	/ [Transcriptional regulator; cupA gene regulator C, CgrC]	Transcriptional regulators	
PA14_37250	*PA14_37250*	-1.86	/ Major facilitator transporter	Transport of small molecules	
PA14_37590	*kynB*	-1.69	KynB / Kynurenine formamidase	Amino acid biosynthesis and metabolism; Putative enzymes	Amino acids
PA14_38500	*PA14_38500*	1.65	/ IclR family transcriptional regulator	Transcriptional regulators	

Locus	Gene	FC[a]	Product /Function[b] [function][c]	Protein Class Assignment[d]	Subclass[d]
PA14_39350	*rbsB*	1.76	/ Ribose ABC transporter substrate-binding protein	Transport of small molecules	
PA14_39460	*PA14_39460*	-1.64	/ [Catalase]	Hypothetical, unclassified, unknown	
PA14_39590	*metE*	-1.98	MetE / 5-methyltetrahydropteroyltriglutamate/ homocysteine S-methyltransferase	Amino acid biosynthesis and metabolism	Amino acids
PA14_40650	*PA14_40650*	-1.83	/ Hypothetical protein [Tsi1-like T6SS immunity protein]	Hypothetical, unclassified, unknown	T6SS-related
PA14_40660	*PA14_40660*	-2.12	/ Hypothetical protein [Tse1-like T6SS effector protein]	Hypothetical, unclassified, unknown	T6SS-related
PA14_41420	*PA14_41420*	-1.57	/ Hypothetical protein	Hypothetical, unclassified, unknown	
PA14_41760	*PA14_41760*	-1.92	/ Hypothetical protein	Hypothetical, unclassified, unknown	
PA14_41790	*PA14_41790*	-1.63	/ Hypothetical protein	Hypothetical, unclassified, unknown	
PA14_42630	*PA14_42630*	1.66	/ Translocation protein in T3SS	Protein secretion/export apparatus	T3SS
PA14_42660	*pscU*	2.91	PscU / Translocation protein in T3SS	Protein secretion/export apparatus	T3SS
PA14_42700	*alkA*	-1.67	AlkA / DNA-3-methyladenine glycosylase II	DNA replication, recombination, modification and repair	
PA14_43610	*PA14_43610*	-1.78	/ AMP-binding protein	Fatty acid and phospholipid metabolism	
PA14_43780	*PA14_43780*	-1.60	/ Oxoreductase	Energy metabolism	
PA14_43790	*PA14_43790*	-1.63	/ Aldehyde dehydrogenase	Putative enzymes	
PA14_44710	*xdhA*	-1.56	XdhA / Xanthine dehydrogenase	Nucleotide biosynthesis and metabolism	
PA14_44800	*PA14_44800*	-1.75	/ Transporter [Xanthine/uracil/vitamin C permease]	Membrane proteins; Transport of small molecules	
PA14_44830	*PA14_44830*	-1.69	/ [Chitin or polysaccharide deacetylase family protein]	Carbon compound catabolism	
PA14_44840	*PA14_44840*	-1.73	/ [possible OHCU decarboxylase]	Hypothetical, unclassified, unknown	
PA14_44850	*alc*	-1.72	Alc / Allantoicase	Carbon compound catabolism	
PA14_44860	*PA14_44860*	-1.69	/ Ureidoglycolate hydrolase	Central intermediary metabolism	

Locus	Gene	FC[a]	Product /Function[b] [function][c]	Protein Class Assignment[d]	Subclass[d]
PA14_44880	*PA14_44880*	-2.43	/ Hypothetical protein	Membrane proteins	
PA14_45350	*ccmC*	1.77	CcmC / Heme exporter protein	Transport of small molecules	Heme
PA14_46370	*PA14_46370*	-1.78	/ Two-component sensor [Sensor histidine kinase/response regulator]	Two-component regulatory systems	
PA14_46640	*PA14_46640*	-2.86	/ Siderophore receptor	Transport of small molecules	Iron utilization
PA14_46910	*PA14_46910*	-1.97	/ ABC transporter substrate-binding protein	Transport of small molecules	Amino acids
PA14_46920	*PA14_46920*	-1.54	/ ABC transporter permease [Glutamate/aspartate transporter GltJ]	Membrane proteins; Transport of small molecules	Amino acids
PA14_46930	*PA14_46930*	-1.52	/ ABC transporter permease [Glutamate/aspartate transporter GltK]	Transport of small molecules	Amino acids
PA14_46950	*PA14_46950*	-1.63	/ ABC transporter ATP-binding protein [Glutamate/aspartate transporter GtlL]	Transport of small molecules	Amino acids
PA14_46960	*ggt*	-1.94	Ggt / Gamma-glutamyltranspeptidase	Central intermediary metabolism	
PA14_46970	*ansB*	-2.29	AnsB / Glutaminase-asparaginase	Amino acid biosynthesis and metabolism	Amino acids
PA14_48060	*aprA*	1.67	AprA / Alkaline metalloproteinase	Secreted factors (toxins, enzymes, alginate)	
PA14_48140	*aprX*	1.86	/ Hypothetical protein [AprX in *P. aeruginosa* PAO1]	Membrane proteins; Secreted factors (toxins, enzymes, alginate)	
PA14_49320	*PA14_49320*	-1.71	/ [Alginate lyase 2]	Hypothetical, unclassified, unknown	
PA14_49560	*toxA*	-1.89	ToxA / Exotoxin A	Secreted factors (toxins, enzymes, alginate)	
PA14_49650	*PA14_49650*	-2.15	/ Hypothetical protein	Hypothetical, unclassified, unknown	
PA14_49850	*PA14_49850*	1.53	/ Hypothetical protein	Hypothetical, unclassified, unknown	
PA14_50610	*PA14_50610*	-1.78	/ Short-chain dehydrogenase	Putative enzymes	
PA14_50620	*PA14_50620*	-1.63	/ Hypothetical protein [possible bacteriophage protein]	Hypothetical, unclassified, unknown	
PA14_50770	*PA14_50770*	1.63	/ Transporter [putative gluconate transporter]	Transport of small molecules	
PA14_51680	*PA14_51680*	2.36	/ Radical activating enzyme [SAM domain]	Putative enzymes	

Locus	Gene	FC[a]	Product /Function[b] [function][c]	Protein Class Assignment[d]	Subclass[d]
PA14_52080	*PA14_52080*	1.71	/ Hypothetical protein	Hypothetical, unclassified, unknown	
PA14_52090	*PA14_52090*	1.84	/ Hypothetical protein	Hypothetical, unclassified, unknown	
PA14_52120	*PA14_52120*	3.20	/ Hypothetical protein	Hypothetical, unclassified, unknown	
PA14_53210	*PA14_53210*	-1.55	/ Hypothetical protein	Hypothetical, unclassified, unknown	
PA14_53260	*PA14_53260*	-1.67	/ Hypothetical protein [threonine dehydratase]	Hypothetical, unclassified, unknown	
PA14_53400	*PA14_53400*	1.72	/ Oxidoreductase	Putative enzymes	
PA14_53410	*PA14_53410*	1.83	/ Transcriptional regulator [TetR family]	Transcriptional regulators	
PA14_53820	*PA14_53820*	1.79	/ [N-acetylmuramoyl-L-alanine amidase]	Antibiotic resistance and susceptibility	
PA14_54130	*PA14_54130*	-1.54	/ Transcriptional regulator [LysR family]	Transcriptional regulators	
PA14_54540	*PA14_54540*	2.92	/ [Tricarboxylate transport protein TctC]	Hypothetical, unclassified, unknown	
PA14_54630	*PA14_54630*	-1.93	/ Acyl-CoA dehydrogenase	Putative enzymes	
PA14_54640	*PA14_54640*	-1.68	/ Enoyl-CoA hydratase	Putative enzymes	
PA14_54750	*PA14_54750*	2.11	/ Hypothetical protein	Hypothetical, unclassified, unknown	
PA14_54850	*PA14_54850*	2.50	/ [Bacteriophage-related integrase]	Hypothetical, unclassified, unknown	
PA14_54870	*PA14_54870*	1.79	/ [Transcriptional regulator, AraC family]	Hypothetical, unclassified, unknown	
PA14_55810	*PA14_55810*	-1.59	/ Two-component response regulator LuxR family (PAO1 PrpB)	Two-component regulatory systems	
PA14_56100	*PA14_56100*	-1.52	/ Hypothetical protein [RDD domain-containing protein]	Membrane proteins	
PA14_56200	*PA14_56200*	-1.60	/ Hypothetical protein [possible lipoprotein]	Fatty acid and phospholipid metabolism	

Locus	Gene	FC[a]	Product /Function[b] [function][c]	Protein Class Assignment[d]	Subclass[d]
PA14_56360	*PA14_56360*	1.65	/ Hypothetical protein [possible arylsulfate sulfotransferase]	Hypothetical, unclassified, unknown	
PA14_58130	*mreC*	1.78	MreC / Rod shape-determining protein	Cell wall / LPS / capsule	
PA14_58240	*PA14_58240*	-1.75	/ Hypothetical protein	Hypothetical, unclassified, unknown	
PA14_58350	*dppA1*	-1.81	DppA1 / Dipeptide-binding ABC transporter substrate-binding protein	Transport of small molecules	
PA14_59390	*PA14_59390*	1.92	/ Hypothetical protein	Hypothetical, unclassified, unknown	
PA14_59780	*rcsC*	1.60	RcsC / Kinase sensor protein	Two-component regulatory systems	
PA14_59970	*PA14_59970*	1.61	/ Hypothetical protein	Hypothetical, unclassified, unknown	
PA14_60030	*PA14_60030*	1.54	/ Hypothetical protein	Hypothetical, unclassified, unknown	
PA14_60490	*PA14_60490*	-1.63	/ Cytochrome c family protein	Energy metabolism	
PA14_60530	*PA14_60530*	1.95	/ Hypothetical protein	Hypothetical, unclassified, unknown	
PA14_60730	*PA14_60730*	-1.67	/ Outer membrane protein	Membrane proteins; Transport of small molecules	
PA14_60750	*pra*	-1.81	Pra / protein activator	Carbon compound catabolism	
PA14_61270	*PA14_61270*	-1.97	/ Hypothetical protein	Hypothetical, unclassified, unknown	
PA14_61920	*PA14_61920*	-3.00	/ Hypothetical protein	Hypothetical, unclassified, unknown	
PA14_61940	*PA14_61940*	-1.89	/ Hypothetical protein	Hypothetical, unclassified, unknown	
PA14_62100	*PA14_62100*	2.92	/ Sulfite oxidase subunit YedZ	Membrane proteins	
PA14_62110	*PA14_62110*	2.77	/ Sulfite oxidase subunit YedY	Putative enzymes	
PA14_62130	*ilvC*	-1.54	IlvC / Ketol-acid reductoisomerase	Amino acid biosynthesis and metabolism; Biosynthesis of cofactors, prosthetic groups and carriers	Amino acids

Locus	Gene	FC[a]	Product /Function[b] [function][c]	Protein Class Assignment[d]	Subclass[d]
PA14_62150	*ilvH*	-1.70	IlvH / Acetolactate synthase 3 regulatory subunit	Amino acid biosynthesis and metabolism; Biosynthesis of cofactors, prosthetic groups and carriers	Amino acids
PA14_62160	*ilvI*	-1.57	IlvI / Acetolactate synthase 3 catalytic subunit	Amino acid biosynthesis and metabolism; Biosynthesis of cofactors, prosthetic groups and carriers	Amino acids
PA14_64350	*ureA*	-1.86	UreA / Urease subunit gamma	Central intermediary metabolism	
PA14_64550	*PA14_64550*	2.25	/ [Thiosulfate reductase cytochrome B subunit]	Energy metabolism	
PA14_64840	*PA14_64840*	-1.70	/ Short-chain dehydrogenase	Putative enzymes	
PA14_64870	*PA14_64870*	-2.33	/ ABC transporter ATP-binding protein [for Branched-chain amino acid transport]	Transport of small molecules	Amino acids
PA14_64950	*pncA*	-2.69	PncA / [Nicotinamidase]	Central intermediary metabolism	
PA14_64960	*pncB1*	-2.09	PncB1 / Nicotinate phosphoribosyl-transferase	Biosynthesis of cofactors, prosthetic groups and carriers	
PA14_64980	*nadE*	-2.25	NadE / NAD synthetase	Amino acid biosynthesis and metabolism;	Amino acids
PA14_65030	*PA14_65030*	1.60	/ [Putative metalloendopeptidase-related membrane protein]	Membrane proteins	
PA14_65630	*PA14_65630*	1.50	/ [Putative aspartyl protease]	Putative enzymes	
PA14_66440	*metY*	-1.52	MetY / O-acetylhomoserine aminocarboxy-propyltransferase	Amino acid biosynthesis and metabolism	Amino acids
PA14_66540	*PA14_66540*	-2.89	/ Hypothetical protein	Hypothetical, unclassified, unknown	
PA14_67350	*hutU*	-1.87	HutU / Urocanate hydratase	Amino acid biosynthesis and metabolism	Amino acids
PA14_67510	*estA*	-1.58	EstA / Esterase	Fatty acid and phospholipid metabolism	
PA14_67620	*PA14_67620*	1.86	/ Hypothetical protein	Hypothetical, unclassified, unknown	
PA14_67640	*PA14_67640*	-1.77	/ Hypothetical protein	Hypothetical, unclassified, unknown	
PA14_67830	*PA14_67830*	-1.59	/ Hypothetical protein	Hypothetical, unclassified, unknown	
PA14_68260	*PA14_68260*	-1.61	/ C4-dicarboxylate-binding protein [DctP family]	Membrane proteins	
PA14_68280	*PA14_6*	-1.74	/ Dicarboxylate transporter [DctQ subunit]	Membrane proteins; Transport of small	

Locus	Gene	FC[a]	Product /Function[b] [function][c]	Protein Class Assignment[d]	Subclass[d]
PA14_68290	*PA14_68290*	-1.54	/ C4-dicarboxylate transporter [DctM subunit]	Membrane proteins	
PA14_69380	*PA14_69380*	-1.51	/ Peptidyl-prolyl cis-trans isomerase, FkbP-type	Chaperones and heat shock proteins; Translation, post-translational modification, degradation	
PA14_70600	*PA14_70600*	-1.88	/ HU family DNA-binding protein	DNA replication, recombination, modification and repair	
PA14_71070	*PA14_71070*	-1.82	/ AraC family transcriptional regulator	Transcriptional regulators	
PA14_71460	*glyA1*	-2.38	GlyA1 / Serine hydroxymethyltransferase	Amino acid biosynthesis and metabolism	Amino acids
PA14_71900	*PA14_71900*	1.60	/ Hypothetical protein	Hypothetical, unclassified, unknown	
PA14_72410	*PA14_72410*	-1.80	/ Multiple antibiotic resistance (MarC-like) protein	Antibiotic resistance and susceptibility	
PA14_72760	*PA14_7*	1.63	/ Beta-lactamase	Adaptation, protection; Antibiotic resistance	
PA14_73040	*amiA*	3.00	AmiA / N-acetylmuramoyl-L-alanine amidase	Cell wall / LPS / capsule	
PA14_73060	*PA14_73060*	2.63	/ Carbonic anhydrase-like protein	Transport of small molecules	

[a]Upregulation (green) and downregulation (red) of gene expression in PA14 grown in blood from SBPs or TPs compared to HVs

[b]Product /Function from UCBPP-PA14 Genome database http://pseudomonas.com/

[c][function from Pseudomonas Ortholog Database] http://pseudoluge.pseudomonas.com/

[d]From the PseudoCap database http://pseudomonas.com/pseudocap

[e]Genes highlighted in yellow were discussed in the text and/or figures
