# Supplementary material for: Pseudomonas aeruginosa Alters Its Transcriptome Related to Carbon Metabolism and Virulence as a Possible Survival Strategy in Blood from Trauma Patients
Source: mSystems. 2019 May 7;4(4):e00312-18. doi: 10.1128/mSystems.00312-18 (PMC6506614; doi:10.1128/mSystems.00312-18)
Supplement: TABLE S3 [file mSystems.00312-18-st003.pdf]

Table S3

Differentially expressed predicted antisense RNA plus large and small RNA products.

Start[a]	Stop[b]	Length	Strand	Product	FC[c] ≥1.5 ≥-1.5	*q* [d] ≤0.05	Comments
64255	63730	525	-	antisense: PA14_00630	2.4	2.8E-25	
328092	327954	138	-	antisense: PA14_03630	-2.3	5.78E-08	
369305	369090	215	-	antisense: PA14_04100	3.0	2.01E-30	
653645	653892	247	+	antisense: PA14_07580	2.1	4.16E-19	
734616	733096	1520	-	antisense: PA14_08570[e]	4.0	2.17E-138	16S rRNA
737953	735100	2853	-	antisense: PA14_08600	6.6	1.04E-291	23S rRNA
765237	764974	263	-	antisense: *rplE*	2.9	5.59E-26	
775136	774767	369	-	**antisense: *bfrA*** [f]	**2.2**	1.12E-17	nc[g]
775296	775611	315	+	antisense: *uvrA*	2.3	4.93E-20	
805283	805500	217	+	**antisense: phzS**	**-2.4**	5.82E-12	1.9
1016946	1016918	28	-	antisense: PA14_11730	-2.9	2.64E-07	
1053400	1053276	124	-	antisense: PA14_12170	3.0	1.03E-15	
1222942	1223625	683	+	antisense: PA14_14340	-54.0	0.00E+00	nc
1274815	1275275	460	+	antisense: *leuA*	3.6	5.35E-55	-2.1
1522104	1521977	127	-	antisense: *rpmE2*	3.8	9.95E-30	
1836723	1837064	341	+	antisense: PA14_21220	6.0	3.89E-86	
1849687	1849567	120	-	antisense: *fadD2*	2.5	3.15E-09	
1863998	1863886	112	-	antisense: PA14_21490	2.0	6.42E-07	
1870523	1870313	210	-	antisense: PA14_21570 PA14_21580	6.0	1.57E-144	3.0 2.8
1902333	1902404	71	+	antisense: *tsp*	6.0	4.75E-46	
1905417	1905055	362	-	**antisense: PA14_21890**	**3.2**	8.70E-48	nc
1962801	1962597	204	-	antisense: PA14_22550	2.2	6.91E-12	
1962801	1962597	204	-	antisense: PA14_22550	2.2	6.91E-12	
2120706	2120697	9	-	antisense: PA14_24360	2.4	6.18E-06	
2153404	2153293	111	-	antisense: PA14_24630	2.0	1.00E-06	
2190534	2190794	260	+	antisense: PA14_25040	2.0	6.52E-11	
2194052	2193777	275	-	antisense: *fadB*	2.3	8.09E-14	
2204351	2204396	45	+	antisense: PA14_25220	2.0	1.45E-03	
2320151	2320248	97	+	antisense: PA14_26640	3.0	7.74E-16	
2356351	2356217	134	-	antisense: PA14_27120	2.0	4.34E-09	
2360088	2360104	16	+	antisense: PA14_27150	-2.3	8.91E-04	
2802689	2803426	737	+	antisense: PA14_32250	4.3	2.45E-204	5.2
3009991	3010452	461	+	antisense: PA14_33870	2.3	3.55E-36	1.8
3084412	3086372	1960	+	antisense: PA14_34720 PA14_34730 PA14_34740	7.1	0.00E+00	
3239166	3239282	116	+	antisense: PA14_36370	2.0	5.16E-06	
3418139	3417966	173	-	antisense: PA14_38340	3.3	3.02E-27	
3484578	3484761	183	+	antisense: PA14_39100	-2.1	4.01E-06	
3518240	3518513	273	+	antisense: PA14_39520	-22.0	0.00E+00	nc
3612543	3612599	56	+	antisense: PA14_40430	2.9	7.30E-09	
3656240	3656520	280	+	antisense: PA14_41010	2.1	1.34E-11	
3686273	3686214	59	-	antisense: PA14_41290	2.2	2.17E-05	
3739392	3739278	114	-	antisense: PA14_41860	2.0	3.96E-07	
3854106	3853766	340	-	antisense: PA14_43250	2.4	6.44E-19	
3924407	3924741	334	+	antisense: PA14_44080	2.1	1.36E-15	

3924510	3924046	464	-	antisense: *gltA*	2.0	1.27E-12	
4049209	4048834	375	-	**antisense: PA14_45470**	**2.0**	1.46E-11	
4492049	4491818	231	-	antisense: *braG*	-2.3	6.21E-08	
4563787	4562873	914	-	**antisense: *mvfR***	**3.0**	6.19E-58	nc
4625023	4624794	229	-	antisense: *lpxO2*	-2.2	1.10E-08	
4695835	4695098	737	-	antisense: PA14_52940	2.0	3.53E-16	
4808699	4809117	418	+	antisense: *pruR*	2.6	6.63E-37	
4952318	4957474	5156	+	antisense: PA14_55631 PA14_55634 PA14_55635 PA14_55637	6.0	3.25E-275	23S rRNA; tRNA-Ala; tRNA-Ile; 16S rRNA
5060140	5060202	62	+	antisense: PA14_56800	-2.3	1.14E-04	
5266416	5266240	176	-	antisense: PA14_59140 PA14_59150	3.3	8.44E-27	
5350100	5349555	545	-	antisense: PA14_60070	3.4	5.17E-74	
5384678	5385115	437	+	antisense: PA14_60390	2.3	8.14E-24	
5399019	5398890	129	-	antisense: PA14_60570	5.5	6.14E-47	
5474606	5474583	23	-	antisense: PA14_61300	-2.2	7.49E-04	
5536325	5539178	2853	+	antisense: PA14_62060	6.6	4.75E-292	23S rRNA
5539662	5541182	1520	+	antisense: PA14_62090	4.0	2.17E-138	16S rRNA
5608393	5608523	130	+	antisense: *ftsH*	4.0	5.86E-32	
5714456	5714335	121	-	antisense: *accC*	2.1	3.04E-07	
6140565	6140680	115	+	antisense: *gcvP1*	2.2	7.33E-10	
6154122	6154310	188	+	antisense: *pepP*	-18.0	0.00E+00	nc
6312796	6315649	2853	+	antisense: PA14_70880	6.6	1.04E-291	23S rRNA
6316133	6317653	1520	+	antisense: PA14_70910	4.0	2.17E-138	16S rRNA
6356929	6356474	455	-	antisense: PA14_71350 PA14_71360	-2.9	3.27E-20	
6382634	6382509	125	-	antisense: *aspA*	-2.8	3.22E-08	
2786835	2787657	822	+	sense: *PA14_32015*[h]	6.6	0.00E+00	CzcA amino terminus
2787806	2789915	2109	+	sense: *PA14_32025*	6.2	0.00E+00	CzcA carboxy terminus
57035	57139	104	+	-	2.3	1.94E-06	
61368	61149	219	-	-	-3.0	1.51E-26	
200534	200500	34	-	-	-2.4	5.66E-04	
226488	226476	12	-	-	-2.3	1.83E-04	
244004	243946	58	-	-	3.0	6.27E-08	
249913	249934	21	+	-	-4.6	1.70E-28	
269220	269202	18	-	-	3.3	1.79E-18	
279717	279566	151	-	-	2.0	1.78E-07	
309605	309748	143	+	-	4.8	7.12E-32	
329965	329956	9	-	-	2.3	1.02E-05	
330918	330785	133	-	-	2.0	2.76E-17	
332114	331962	152	-	-	2.5	2.68E-48	
374503	374521	18	+	-	2.7	8.99E-09	
377253	377028	225	-	-	-3.6	1.89E-19	

384642	384575	67	-	-	-2.6	2.50E-05
403447	403506	59	+	-	2.1	1.72E-07
458075	457976	99	-	-	3.0	3.46E-14
481583	481492	91	-	-	-2.3	2.65E-05
514654	514466	188	-	-	2.3	1.20E-21
564678	564698	20	+	-	-2.8	1.51E-07
565458	565533	75	+	-	-5.7	4.86E-41
565783	565792	9	+	-	-2.6	3.94E-08
610279	610210	69	-	-	2.4	2.41E-09
634868	634779	89	-	-	2.3	3.78E-08
673111	673135	24	+	-	3.0	7.68E-11
784463	784623	160	+	-	-2.0	3.05E-06
799927	800011	84	+	-	-2.8	3.88E-54
802613	802792	179	+	-	-2.5	2.84E-09
805206	805216	10	+	-	-2.1	3.49E-04
806855	806528	327	-	-	5.0	7.65E-100
807525	807504	21	-	-	6.0	1.15E-63
808375	808363	12	-	-	2.0	6.25E-05
813422	813136	286	-	-	2.3	6.65E-23
840541	840449	92	-	-	3.8	2.89E-21
906820	906724	96	-	-	4.8	1.12E-29
908551	908647	96	+	-	3.1	2.23E-18
933885	933825	60	-	-	3.8	1.30E-50
1042855	1042885	30	+	-	2.4	4.88E-05
1107375	1107457	82	+	-	2.3	4.16E-08
1117707	1117870	163	+	-	2.0	1.01E-14
1210068	1210133	65	+	-	2.0	7.89E-05
1310747	1310736	11	-	-	2.1	2.76E-04
1334440	1334545	105	+	-	2.8	3.02E-11
1349852	1349837	15	-	-	-2.0	4.94E-03
1384119	1384136	17	+	-	-2.1	2.22E-05
1437572	1437554	18	-	-	2.2	5.02E-04
1455960	1455934	26	-	-	-3.8	1.01E-16
1477571	1477600	29	+	-	2.8	2.65E-07
1492951	1493022	71	+	-	-2.2	4.88E-03
1505272	1505293	21	+	-	-2.0	4.32E-03
1545244	1545205	39	-	-	-2.1	2.23E-03
1561325	1561359	34	+	-	3.6	3.31E-18
1597096	1596991	105	-	-	2.0	2.51E-06
1599848	1599983	135	+	ReaL	2.0	4.03E-09
1635661	1635234	427	-	-	-2.1	1.04E-22
1648377	1648572	195	+	-	2.1	1.11E-20
1676713	1676674	39	-	-	2.1	3.94E-04
1685556	1685652	96	+	-	2.8	2.66E-46
1689131	1689248	117	+	-	3.2	6.16E-38
1699602	1699715	113	+	-	2.0	7.38E-05
1725316	1725523	207	+	-	-3.2	2.00E-28
1736707	1736732	25	+	-	-2.6	3.18E-05
1749126	1749177	51	+	-	7.4	7.42E-94
1750183	1750249	66	+	-	14.5	0.00E+00
1870104	1870121	17	+	-	2.7	3.52E-15
1872668	1872899	231	+	-	3.2	1.40E-64

1887235	1887202	33	-	-	2.2	1.06E-04
1934976	1934915	61	-	-	3.6	4.10E-17
1978119	1978012	107	-	-	2.0	3.37E-07
1983463	1983606	143	+	-	2.1	1.46E-11
1993611	1993668	57	+	-	-2.0	1.59E-03
2025890	2025947	57	+	-	2.7	6.33E-13
2045697	2045716	19	+	-	-3.3	6.61E-13
2253608	2253666	58	+	-	2.1	1.80E-05
2275154	2275079	75	-	-	-2.1	4.22E-05
2303834	2303550	284	-	-	2.5	4.38E-29
2362094	2362489	395	+	-	2.7	6.32E-32
2388350	2388427	77	+	-	2.1	8.61E-22
2411667	2411678	11	+	-	5.3	1.59E-44
2411892	2411937	45	+	-	7.4	1.31E-85
2417927	2417943	16	+	-	2.1	1.37E-04
2423522	2423639	117	+	-	3.6	5.52E-17
2436434	2436421	13	-	-	-2.1	7.44E-05
2455743	2455780	37	+	-	-4.0	1.68E-22
2492542	2492412	130	-	-	2.7	4.14E-14
2538176	2538038	138	-	-	-2.3	3.94E-11
2644422	2644482	60	+	-	3.6	2.90E-15
2667798	2667876	78	+	-	2.3	4.39E-16
2670884	2670869	15	-	-	2.2	2.17E-04
2730130	2730149	19	+	-	-2.6	3.91E-05
2745826	2745686	140	-	-	-2.2	5.98E-06
2757997	2758085	88	+	-	-2.2	3.31E-14
2783778	2783529	249	-	-	4.7	1.62E-81
2785328	2785379	51	+	-	9.5	1.26E-115
2802845	2802731	114	-	-	7.8	1.27E-138
2888995	2889136	141	+	-	-2.9	8.00E-18
2908551	2908568	17	+	-	2.3	2.05E-05
2912026	2912098	72	+	-	-2.0	1.84E-11
2925194	2925332	138	+	-	2.0	2.31E-08
2948952	2948991	39	+	-	-2.6	4.03E-20
3014294	3014188	106	-	-	2.4	2.95E-09
3038665	3038697	32	+	-	2.6	8.13E-07
3058178	3058157	21	-	-	6.8	5.38E-85
3059330	3059292	38	-	-	10.3	1.01E-245
3060174	3060165	9	-	-	16.0	0.00E+00
3062547	3062494	53	-	-	7.6	2.21E-117
3075621	3075597	24	-	-	2.6	6.85E-06
3216785	3216766	19	-	-	-4.5	2.93E-21
3224444	3224524	80	+	-	-2.2	5.54E-04
3230291	3230204	87	-	-	2.2	4.62E-11
3232595	3232552	43	-	-	2.6	3.95E-10
3236469	3236397	72	-	-	2.6	8.67E-16
3246608	3246619	11	+	-	4.5	1.87E-39
3249928	3249796	132	-	-	2.5	4.57E-14
3318288	3318311	23	+	-	-2.4	5.12E-04
3319542	3319571	29	+	-	-2.7	4.38E-05
3347302	3347402	100	+	-	-3.8	1.79E-13
3426030	3426062	32	+	-	3.3	2.54E-12

3441642	3441742	100	+	-	2.1	9.54E-09
3512853	3513092	239	+	-	-2.1	2.17E-07
3546312	3546402	90	+	-	2.4	2.15E-10
3556128	3556107	21	-	-	6.0	1.15E-63
3556978	3556966	12	-	-	2.0	6.25E-05
3630810	3630859	49	+	-	-2.1	8.32E-03
3738075	3738049	26	-	-	2.0	2.38E-07
3897277	3897483	206	+	-	-2.0	5.09E-12
3959448	3959561	113	+	-	2.7	1.07E-14
3990202	3990270	68	+	-	-2.9	1.13E-08
3992674	3992782	108	+	-	-2.2	5.52E-10
3997582	3997635	53	+	-	-3.5	8.01E-19
4014792	4014865	73	+	-	-3.5	1.35E-17
4027326	4027728	402	+	-	-2.1	1.90E-10
4146755	4146825	70	+	-	2.1	5.19E-05
4178838	4179010	172	+	-	-2.4	2.66E-24
4182929	4182999	70	+	-	-2.1	1.11E-18
4198208	4197971	237	-	-	2.5	1.12E-47
4279322	4279182	140	-	-	2.0	4.72E-10
4285964	4286194	230	+	-	-5.6	7.20E-172
4401009	4401134	125	+	-	2.0	1.87E-07
4417716	4417839	123	+	-	-2.0	3.29E-04
4428274	4428241	33	-	-	2.1	5.57E-05
4485828	4485653	175	-	-	2.0	7.69E-12
4491974	4492001	27	+	-	-3.3	9.43E-10
4568030	4568008	22	-	-	2.0	1.23E-15
4571549	4571522	27	-	-	2.1	5.20E-04
4736602	4736563	39	-	-	2.0	1.82E-09
4743227	4743250	23	+	-	3.2	1.61E-12
4774596	4774449	147	-	-	-3.7	1.49E-36
4787163	4787256	93	+	-	-2.3	2.20E-03
4801008	4801020	12	+	-	-3.1	4.66E-12
4806518	4806629	111	+	-	2.4	1.03E-15
4835040	4835068	28	+	-	2.1	1.45E-04
4861607	4861446	161	-	-	2.6	4.34E-23
4888811	4888903	92	+	-	-2.6	2.41E-31
4900204	4900284	80	+	-	-3.1	8.02E-11
5007181	5007215	34	+	-	-2.3	2.50E-06
5029936	5029844	92	-	-	-3.3	8.21E-11
5047121	5047156	35	+	-	-2.2	6.60E-03
5119903	5120244	341	+	-	2.0	1.11E-20
5174418	5174330	88	-	-	2.6	1.58E-14
5177144	5177056	88	-	-	-2.6	1.03E-05
5264754	5264995	241	+	-	2.0	5.85E-11
5292645	5292764	119	+	-	-2.1	4.92E-09
5347847	5347968	121	+	-	-2.0	2.01E-05
5370203	5370018	185	-	-	3.2	8.81E-24
5388487	5388379	108	-	-	2.1	5.86E-10
5398440	5398591	151	+	-	2.1	3.34E-10
5521532	5521511	21	-	-	-2.4	4.52E-04
5556009	5555936	73	-	PrrF1	9.3	1.48E-113
5563443	5563544	101	+	-	3.6	3.07E-23

5654193	5654308	115	+	-	2.2	4.30E-12
5673607	5673586	21	-	-	-4.7	5.72E-35
5732048	5732159	111	+	-	-3.0	3.50E-07
5739000	5738964	36	-	-	3.6	1.71E-22
5749614	5749550	64	-	-	6.6	1.21E-58
5789169	5789188	19	+	-	-3.1	5.85E-12
6010689	6010573	116	-	-	-2.5	4.80E-05
6012155	6012094	61	-	-	-2.8	2.76E-07
6015331	6015225	106	-	-	-3.0	6.09E-29
6017106	6017012	94	-	-	-2.1	4.84E-05
6036887	6036877	10	-	-	2.3	2.35E-05
6043146	6043189	43	+	-	-2.6	1.77E-05
6059019	6058871	148	-	-	-2.1	3.03E-10
6224090	6224069	21	-	-	3.0	4.54E-12
6258847	6258866	19	+	-	2.2	1.11E-04
6298198	6298223	25	+	-	3.6	1.04E-19
6348125	6348178	53	+	-	-3.5	6.27E-11
6348782	6348796	14	+	-	-2.5	2.11E-05
6372966	6373058	92	+	-	-2.6	1.87E-12
6374602	6374807	205	+	-	-2.2	4.66E-08
6382150	6382164	14	+	-	-2.6	5.22E-07
6460839	6461078	239	+	-	2.8	1.94E-26

[a]Transcriptional start site

[b]Transcriptional stop site

[c]Each fold change value represents the average expression of each RNA in PA14 grown in the blood of the TPs/HvS; upregulated RNAs, red; downregulated RNAs, green.

[d]*q* value of HVs versus TPs in scientific notation as all values are 0.00 in numeric form.

[e]Yellow-highlighted asRNAs are discussed in the text.

[f]Bolded transcripts were also found in the 2018 study by Eckweiler and Häussler.

[g]Change in gene expression; red, upregulated; green, downregulated; nc, no significant change.

[h]Transcripts encoding the potential amino and carboxy termini of CzcA are highlighted in blue.
