# Supplementary material for: Pseudomonas aeruginosa Alters Its Transcriptome Related to Carbon Metabolism and Virulence as a Possible Survival Strategy in Blood from Trauma Patients
Source: mSystems. 2019 May 7;4(4):e00312-18. doi: 10.1128/mSystems.00312-18 (PMC6506614; doi:10.1128/mSystems.00312-18)
Supplement: TABLE S4 [file mSystems.00312-18-st004.pdf]

Table S4

PA14 genes differentially expressed in blood from severely burned patients (SBPs) and TPs.

Locus	Gene[b]	Product	Fold-Change[a]	
			SBPs	TPs
**PA14_02570[c]**	***mdcC***	**malonate decarboxylase subunit delta**	**11.63**	**6.00**
**PA14_02550**	***mdcA***	**malonate decarboxylase subunit alpha**	**12.13**	**5.86**
**PA14_02560**		**triphosphoribosyl-dephospho-CoA synthase**	**20.73**	**4.20**
**PA14_02590**	***mdcE***	**malonate decarboxylase subunit gamma**	**8.54**	**4.00**
**PA14_02580**	***mdcD***	**malonate decarboxylase subunit beta**	**13.17**	**3.00**
PA14_33860	*lldA*	L-lactate dehydrogenase	3.65	1.85
PA14_58130	*mreC*	rod shape-determining protein MreC	2.53	1.78
PA14_45350	*ccmC*	heme exporter protein CcmC	5.37	1.77
PA14_38500		IclR family transcriptional regulator	7.23	1.65
PA14_49850		hypothetical protein	3.63	1.53
PA14_17340	*ispD*	2-C-Methyl-D-erythritol 4-phosphate cytidylyltransferase	2.94	1.52
PA14_46930		ABC transporter permease	-4.28	-1.52
PA14_66440	*metY*	O-acetylhomoserine aminocarboxypropyltransferase	-4.75	-1.52
**PA14_62130**	***ilvC***	**ketol-acid reductoisomerase**	**-2.77**	**-1.54**
PA14_46920		ABC transporter permease	-3.27	-1.54
PA14_33520		thioesterase	-2.12	-1.55
**PA14_33680**	***fpvA***	**ferripyoverdine receptor**	**-4.34**	**-1.56**
PA14_44710	*xdhA*	xanthine dehydrogenase	-4.43	-1.56
PA14_27520		glutathione peroxidase	-4.16	-1.56
PA14_67510	*estA*	esterase EstA	-2.20	-1.58
PA14_17930	*glpD*	glycerol-3-phosphate dehydrogenase	-11.76	-1.60
**PA14_26020**		**aminopeptidase**	**-4.08**	**-1.63**
PA14_46950		ABC transporter ATP-binding protein	-5.37	-1.63
PA14_17720		LuxR family transcriptional regulator	-2.93	-1.67
PA14_37590	*kynB*	kynurenine formamidase, KynB	-2.06	-1.69
PA14_44860		ureidoglycolate hydrolase	-3.57	-1.69
PA14_44830		hypothetical protein	-5.50	-1.69
**PA14_62150**	***ilvH***	**acetolactate synthase 3 regulatory subunit**	**-2.35**	**-1.70**
PA14_17980	*glpF*	glycerol uptake facilitator protein	-6.06	-1.71
PA14_49320		hypothetical protein	-2.76	-1.71
**PA14_33720**	***pvdN***	**protein PvdN**	**-2.45**	**-1.72**
PA14_44850	*alc*	allantoicase	-3.89	-1.72
PA14_44840		hypothetical protein	-5.33	-1.73
PA14_02260		two-component response regulator	-2.09	-1.73

PA14_16840		lipoprotein	-2.09	-1.74
PA14_50610		short chain dehydrogenase	-2.13	-1.78
PA14_27100	*lipA*	lactonizing lipase	-2.00	-1.78
PA14_71070		AraC family transcriptional regulator	-2.27	-1.82
**PA14_33710**	***pvdO***	**protein PvdO**	**-4.71**	**-1.83**
PA14_37250		major facilitator transporter	-8.50	-1.86
**PA14_33700**	***pvdF***	**pyoverdine synthetase F**	**-4.94**	**-1.89**
PA14_29330		hypothetical protein	-5.47	-1.91
PA14_13140		hypothetical protein	-2.17	-1.93
PA14_54630		acyl-CoA dehydrogenase	-2.14	-1.93
**PA14_46960**	***ggt***	**gamma-glutamyltranspeptidase**	**-3.88**	**-1.94**
PA14_46910		ABC transporter substrate-binding protein	-5.63	-1.97
PA14_34050		hypothetical protein	-2.57	-2.00
**PA14_33690**	***pvdE***	**pyoverdine biosynthesis protein PvdE**	**-2.36**	**-2.11**
**PA14_33810**	***pvdA***	**L-ornithine N5-oxygenase**	**-3.81**	**-2.27**
PA14_20010	*hasR*	heme uptake outer membrane receptor HasR	-11.80	-2.27
**PA14_46970**	***ansB***	**glutaminase-asparaginase**	**-5.99**	**-2.29**
PA14_32905		hypothetical protein	-4.56	-2.32
PA14_02520		hypothetical protein	-6.35	-2.34
**PA14_33500**	***pvdH***	**diaminobutyrate--2-oxoglutarate aminotransferase**	**-4.61**	**-2.35**
PA14_44880		hypothetical protein	-2.89	-2.43
PA14_34030		hypothetical protein	-2.64	-2.67
PA14_25980	*aroF*	phospho-2-dehydro-3-deoxyheptonate aldolase	-2.18	-2.90
PA14_06420		hypothetical protein	-8.60	-3.09
PA14_06430		hypothetical protein	-15.29	-3.15
PA14_34370		ABC maltose/mannitol transporter ATP-binding protein	-2.80	9.60
PA14_21610	*oprO*	pyrophosphate-specific outer membrane porin OprO precursor	-41.78	4.92
PA14_20390	*phnJ*	hypothetical protein	-175.14	4.75
PA14_21600		hypothetical protein	-2.20	4.18
PA14_19530		NAD(P)H-dependent FMN reductase	-164.25	3.11
PA14_13010		hypothetical protein	-27.50	3.08
PA14_73040	*amiA*	N-acetylmuramoyl-L-alanine amidase	-12.59	3.00
PA14_21570		hypothetical protein	-3.04	2.99
PA14_19680		hypothetical protein	-20.75	2.67
PA14_73060		hypothetical protein	-11.19	2.63
PA14_19490		antioxidant protein	-215.29	2.37
PA14_19100	*rhlA*	rhamnosyltransferase chain A	-4.02	2.31
PA14_03710	-	hypothetical protein	-139.59	2.04

PA14_03700	*sbp*	sulfate-binding protein	-65.56	2.02
PA14_07355	-	hypothetical protein	-2.79	1.93
PA14_59390	-	hypothetical protein	-2.31	1.92
PA14_15350		integrase	-2.44	1.88
PA14_48140		hypothetical protein	-16.37	1.86
PA14_31580		acyl-CoA dehydrogenase	-3.61	1.83
PA14_36290		hypothetical protein	-4.43	1.82
PA14_53820		hypothetical protein	-2.28	1.79
PA14_53400		oxidoreductase	-2.04	1.72
**PA14_48060**	***aprA***	**alkaline metalloproteinase**	**-4.71**	**1.67**
PA14_56360		hypothetical protein	-8.61	1.65
PA14_59970		hypothetical protein	-2.77	1.61
PA14_30800		hypothetical protein	-5.86	1.60
PA14_71900		hypothetical protein	-4.07	1.60
PA14_34600		glyceraldehyde-3-phosphate dehydrogenase	-2.36	1.52
PA14_02530		hypothetical protein	-29.81	1.50
PA14_19020		hypothetical protein	2.14	-1.52
PA14_06920		class III pyridoxal phosphate-dependent aminotransferase	3.07	-1.64
PA14_28010		hypothetical protein	3.50	-1.68
**PA14_33010**	***glyA2***	**serine hydroxymethyltransferase**	**9.00**	**-1.73**
PA14_68280		dicarboxylate transporter	2.36	-1.74
PA14_46370		two-component sensor	2.29	-1.78
PA14_40650		hypothetical protein	2.25	-1.83
PA14_67350	*hutU*	urocanate hydratase	11.19	-1.87
PA14_39590	*metE*	5-methyltetrahydropteroyltriglutamate/homocysteine S-methyltransfe[illegible]	9.45	-1.98
PA14_28390		hypothetical protein	3.22	-2.00
**PA14_33030**	***sdaA***	**L-serine dehydratase**	**2.63**	**-2.00**
PA14_14290		hypothetical protein	2.56	-2.19

[a]Upregulation (green) and downregulation (red) of gene expression in PA14 grown in blood from SBPs or TPs compared to healthy volunteeers.

[b]If gene name was available.

[c]Genes in bold are also found in Table 2 in the manuscript.
